# Neutrophils in nasal polyps exhibit transcriptional adaptation and proinflammatory roles that depend on local polyp milieu

**DOI:** 10.1172/jci.insight.184739

**Published:** 2024-11-22

**Authors:** Chen Zhang, Qianqian Zhang, Jiani Chen, Han Li, Fuying Cheng, Yizhang Wang, Yingqi Gao, Yumin Zhou, Le Shi, Yufei Yang, Juan Liu, Kai Xue, Yaguang Zhang, Hongmeng Yu, Dehui Wang, Li Hu, Huan Wang, Xicai Sun

**Affiliations:** 1 ENT Institute and Department of Otorhinolaryngology and; 2 High Altitude Rhinology Research Center, Eye and ENT Hospital, Fudan University, Shanghai, China.; 3 Department of Otolaryngology, Shigatse People’s Hospital, Shigatse City, China.; 4 Med-X Institute, Center for Immunological and Metabolic Diseases, The First Affiliated Hospital of Xi’an JiaoTong University, Xi’an JiaoTong University, Xi’an, Shaanxi, China.

**Keywords:** Inflammation, Neutrophils

## Abstract

Chronic rhinosinusitis with nasal polyps (CRSwNP) is an inflammatory upper airway disease, divided into eosinophilic CRSwNP (eCRSwNP) and noneosinophilic CRSwNP (neCRSwNP) according to eosinophilic levels. Neutrophils are major effector cells in CRSwNP, but their roles in different inflammatory environments remain largely unclear. We performed an integrated transcriptome analysis of polyp-infiltrating neutrophils from patients with CRSwNP, using healthy donor blood as a control. Additional experiments, including flow cytometry and in vitro epithelial cell and fibroblast culture, were performed to evaluate the phenotypic feature and functional role of neutrophils in CRSwNP. Single-cell RNA-sequencing analysis demonstrated that neutrophils could be classified into 5 functional subsets, with GBP5^+^ neutrophils occurring mainly in neCRSwNP and a high proportion of CXCL8^+^ neutrophils in both subendotypes. GBP5^+^ neutrophils exhibited significant IFN-I pathway activity in neCRSwNP. CXCL8^+^ neutrophils displayed increased neutrophil activation scores and mainly secreted oncostatin M (OSM), which facilitates communication with other cells. In vitro experiments showed that OSM enhanced IL-13– or IL-17–mediated immune responses in nasal epithelial cells and fibroblasts. Our findings indicate that neutrophils display transcriptional plasticity and activation when exposed to polyp tissue, contributing to CRSwNP pathogenesis by releasing OSM, which interacts with epithelial cells and fibroblasts depending on the inflammatory environment.

## Abstract 2

Neutrophils exhibited transcriptional plasticity and activation by releasing OSM to interact with epithelial cells and fibroblasts in a manner dependent on the inflammatory milieu.

## Introduction

Chronic rhinosinusitis with nasal polyps (CRSwNP) is a common disease characterized by chronic mucosal inflammation in the upper airway and generally classified as eosinophilic CRSwNP (eCRSwNP) or noneosinophilic CRSwNP (neCRSwNP) based on the eosinophilic status of the nasal polyp (NP) tissue (1, 2). Patients with CRSwNP in Western countries are characterized mainly by type 2 inflammation, with increased eosinophil infiltration and elevated type 2 cytokines. Although the proportion of eCRSwNP in Asian CRSwNP patients has increased in recent years, neCRSwNP still accounts for a relatively high percentage (30%–50%) and is associated with mixed inflammation, including type 1 (IFN-γ) and/or type 3 (IL-17) immune responses (3–5).

Early studies demonstrated prominent neutrophilic inflammation in neCRSwNP patients, whereas accumulating evidence showed increased infiltration of neutrophils in eCRSwNP patients (6). Moreover, the concurrent increase in neutrophil infiltration in eCRSwNP patients is associated with worse quality of life, greater symptom burden, and greater refractoriness (7, 8). A recent study revealed that neutrophil extracellular traps are abundant near epithelial cells in NPs and can induce epithelial basal cell hyperplasia and possibly further polyp formation (9). Additionally, neutrophils in neCRSwNP patients may contribute to tissue fibrosis via TGF-β2 production (10). Despite these findings, the role of neutrophil infiltration in CRSwNP pathogenesis remains largely unknown. In particular, the phenotype and functional differences in neutrophils under the distinct inflammatory milieu of CRSwNP have not been compared.

Single-cell RNA sequencing (scRNA-seq) has emerged as a powerful tool for determining the functional status and heterogeneity of various cells. Although technical difficulties exist in isolating and preserving neutrophils, a few groups have recently applied scRNA-seq to examine the phenotypic and functional heterogeneity of blood and tissue neutrophils in healthy and diseased states (11–14). A systematic analysis of the transcriptomic features of neutrophils from eCRSwNP and neCRSwNP patients is essential for understanding neutrophil plasticity and improving CRSwNP treatment. Here, we firstly performed an integrated transcriptome analysis of CRSwNP neutrophils at the single-cell level to assess the activation status and transcriptome heterogeneity of NP-infiltrating neutrophils in different inflammatory backgrounds.

## Results

### The scRNA-seq profiling maps of neutrophils in NPs.

**Figure 1:**
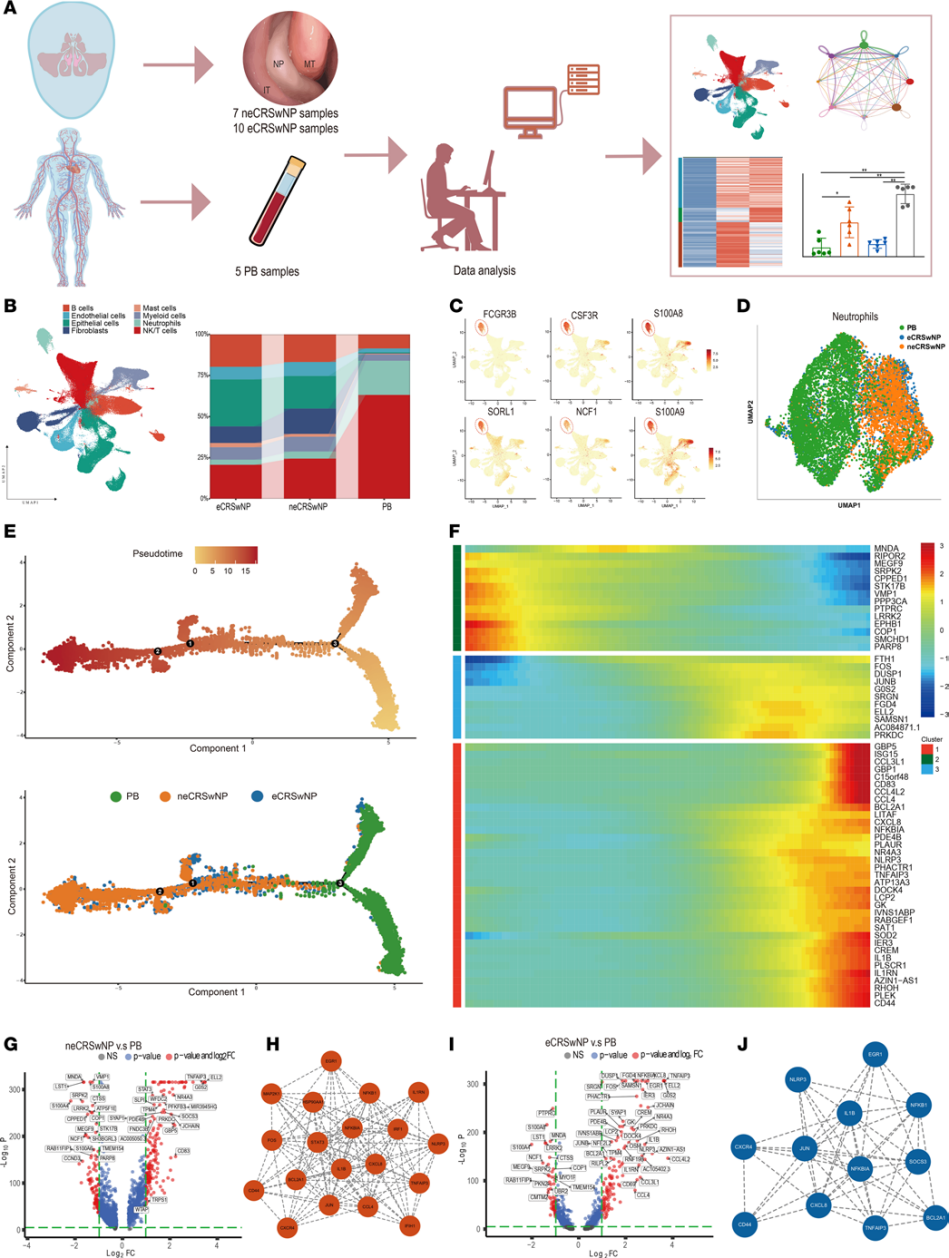
scRNA-seq profiling maps the heterogeneity of neutrophils in nasal polyps. (**A**) Graphical scheme describing the experimental workflow. (**B**) UMAP plot depicting the major cell types identified by scRNA-seq; bar plot depicts the proportion of cell subsets. (**C**) UMAP plots displaying the marker gene expression of neutrophils. (**D**) UMAP plot depicting the neutrophils by groups. (**E**) Trajectory of neutrophils along pseudotime in a 2-dimensional space. Each data point corresponds to a single cell. (**F**) Heatmap showing the dynamic gene expression changes over pseudotime. The differentially expressed genes were clustered hierarchically into 3 groups. (**G**) Volcano plot showing changes in the neCRSwNP neutrophils compared with the PB neutrophils. (**H**) The core network calculated by MCODE in the protein-protein interaction (PPI) network for upregulated genes in the neCRSwNP neutrophils compared with the PB neutrophils. Score = 13.000. (**I**) Volcano plot exhibiting changes in eCRSwNP neutrophils compared with PB neutrophils. (**J**) The core network calculated by MCODE in the PPI network for upregulated genes in eCRSwNP neutrophils compared with PB neutrophils. Score = 8.909.

We constructed a single-cell transcriptome atlas via the integrated analysis and batch effect removal of 22 patient specimens from the NCBI Gene Expression Omnibus (GEO) and Genome Sequence Archive (GSA) databases, along with our own sequencing data (Figure 1A and Supplemental Figure 1, A and B; supplemental material available online with this article; https://doi.org/10.1172/jci.insight.184739DS1). We annotated 8 cell types within distinct clusters according to cell type–specific gene markers (Figure 1, B and C, Supplemental Figure 1C, and Supplemental Table 6).

To assess the role of neutrophils in CRSwNP, 9,735 neutrophils were further analyzed (Figure 1D). We explored neutrophil differentiation trajectories, revealing a unidirectional trajectory from peripheral blood (PB) to locally infiltrated neutrophils (Figure 1E and Supplemental Table 7). Pseudotime heatmap analysis showed heightened expression of genes linked to neutrophil activation (*CXCL8* and *IL1B*) and IFN pathways (*GBP5* and *IGS15*) during later differentiation stages (Figure 1F), indicating functional changes as neutrophils progressed.

Next, we performed differentially expressed gene (DEG) and functional analyses of neutrophils between CRSwNP and PB (Figure 1, G and I, and Supplemental Tables 8 and 9). With Molecular Complex Detection (MCODE) applied for module analysis in the protein-protein interaction (PPI) network, all nodes were classified and colored according to their function. The most compact MCODE module, comprising genes upregulated in both neCRSwNP and eCRSwNP neutrophils compared with PB neutrophils, primarily involved inflammatory and cytokine pathway responses (Figure 1, H and J). Functional and pathway analyses revealed enrichment of cytokine-mediated signaling and response to LPS in both neCRSwNP and eCRSwNP neutrophils (Supplemental Figure 1, F and G, and Supplemental Tables 10–15).

### Neutrophils are activated in both eCRSwNP and neCRSwNP.

**Figure 2:**
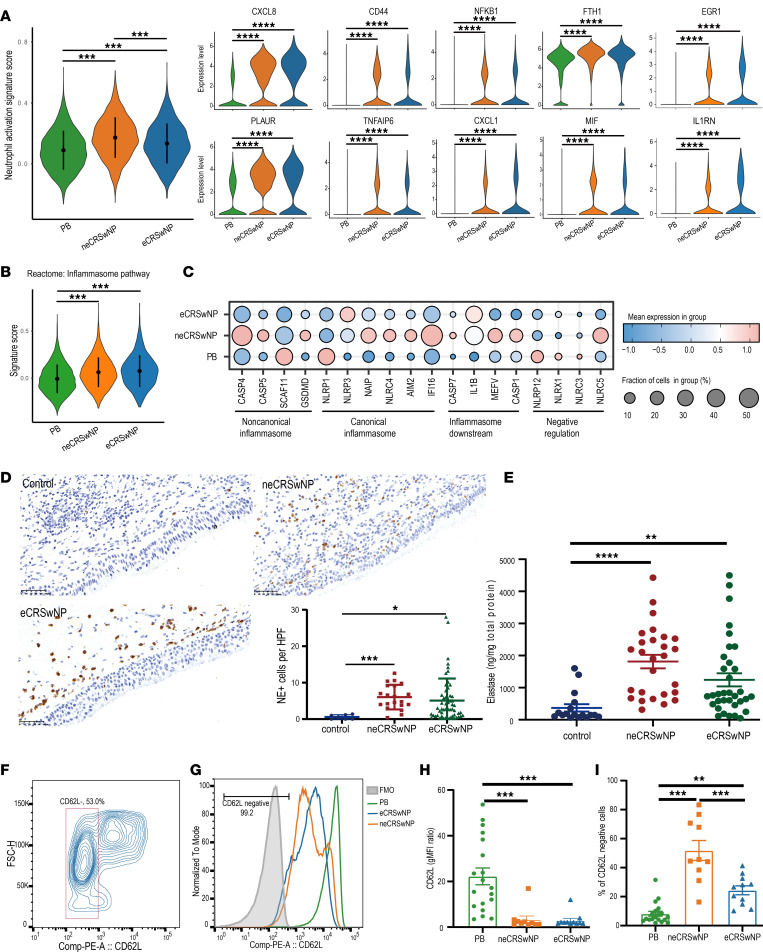
Neutrophils are activated in both eCRSwNP and neCRSwNP. (**A**) Violin plots showing the signature score of the neutrophil activation (GO: 0042119) pathway and the expression levels of pathway-related genes in each group of neutrophils. (**B**) Violin plot showing the signature score of the inflammasome pathway in each group of neutrophils. (**C**) Dot plot depicting the expression levels of inflammasome components in each group of neutrophils. (**D**) Representative images of neutrophil elastase (NE) immunohistochemical staining under high magnification were selected, and the number of NE-positive cells was quantified in high-power fields (HPFs) and evaluated with the Kruskal-Wallis test with Dunn’s post hoc test (control, *n* = 7; neCRSwNP, *n* = 20; eCRSwNP, *n* = 55). Scale bars: 50 μm. (**E**) Scatter plot depicting the NE expression level of tissue homogenates in the control uncinate tissues (UTs) (*n* = 17), neCRSwNP (*n* = 27), and eCRSwNP (*n* = 34) groups evaluated with the Kruskal-Wallis test with Dunn’s post hoc test. (**F**) Representative flow cytometry plot showing the activated neutrophils (CD62L^–^) within the live CD45^+^CD66B^+^CD16^+^ population. (**G**) Representative histogram of flow cytometry showing the levels of CD62L by geometric mean fluorescence intensity (gMFI). (**H**) Graph presenting the differences in the CD62L gMFI ratio on neutrophils evaluated with the Kruskal-Wallis test with Dunn’s post hoc test (PB, *n* = 19; neCRSwNP, *n* = 10; eCRSwNP, *n* = 11). (**I**) Graph presenting the frequency of CD62^–^ neutrophils evaluated with the Kruskal-Wallis test with Dunn’s post hoc test (PB, *n* = 19; neCRSwNP, *n* = 10; eCRSwNP, *n* = 11). *P < 0.05; **P < 0.01; ***P < 0.001; ****P < 0.001.

Neutrophil activation was previously demonstrated by bulk transcriptome sequencing in CRSwNP patients from Western countries (6). Similarly, our scRNA-seq analysis revealed that the neutrophil activation (Gene Ontology [GO] term: 0042119) score was significantly greater in the eCRSwNP group than the PB group (Figure 2A, *P* < 0.001). Additionally, the neCRSwNP group exhibited a greater activation score than both the PB and eCRSwNP groups (*P* < 0.001). Notably, in neCRSwNP and eCRSwNP neutrophils, key activated molecules, such as *CXCL1*, *CXCL8*, *CD44*, *PLAUR*, *NFKB1*, *FTH1*, *TNFAIP6*, and *IL1RN* were upregulated (Figure 2A) (6, 15). In addition, the inflammasome pathway signature score was elevated in neCRSwNP and eCRSwNP neutrophils, indicating the activation of innate inflammatory responses (Figure 2B). Our DEG analysis also revealed increased expression of elements in both canonical and noncanonical inflammasome pathways (Figure 2C).

We next confirmed the increased accumulation of neutrophils in both neCRSwNP and eCRSwNP by immunohistochemistry and ELISA for neutrophil elastase (NE) (Figure 2, D and E). To further validate the activation status of neutrophils in NPs, we used flow cytometry to assess the cell surface expression of CD62L, a marker of neutrophil activation (6, 16) (Figure 2, F and G, and Supplemental Figure 2B). Consistent with a prior study (6), we also detected a decrease in CD62L expression on NP neutrophils (*P* < 0.001, Figure 2H). Furthermore, we observed an increase in the CD62L-negative cell population in eCRSwNP neutrophils compared with that in PB neutrophils (*P* < 0.01), with a more pronounced increase in neCRSwNP neutrophils than in both eCRSwNP and PB neutrophils (*P* < 0.001, Figure 2I). Collectively, our findings indicated that neutrophils are activated and serve a proinflammatory role in CRSwNP.

### Neutrophils in NPs consist of distinct transcriptional subsets.

**Figure 3:**
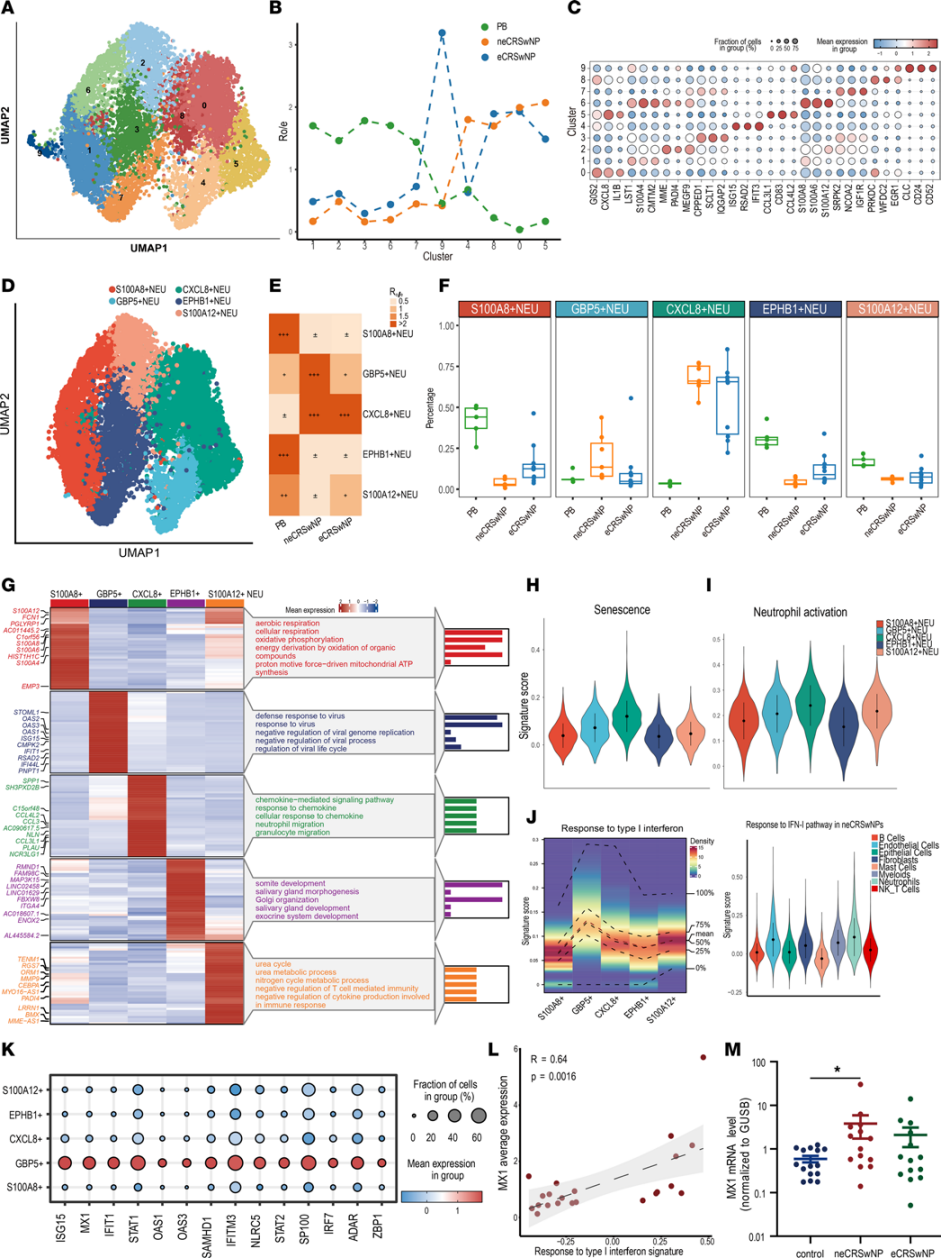
Neutrophils in nasal polyps consist of distinct transcriptional subsets. (**A**) The UMAP plot depicting 10 clusters of neutrophils. (**B**) Line graph presenting the ratio of observed to expected cell numbers (Ro/e) for each cluster. (**C**) Dot plot depicting the top 3 genes in each neutrophil cluster. (**D**) UMAP plot depicting 5 subsets of neutrophils. (**E**) Heatmap showing the Ro/e level of each subset. (**F**) Box-and-whisker plot depicting the proportions of neutrophil subsets in different groups. Shown are the median (line within box), IQR (box bounds), and the range (whiskers); data points beyond whiskers are outliers. (**G**) Heatmap visualizing the 40 genes with the highest expression levels and pathway enrichment for each neutrophil subset. (**H**) The violin plot showing the signature score of the senescence pathway in different neutrophil subsets. (**I**) The violin plot showing the signature score of the neutrophil activation pathway in different neutrophil subsets. (**J**) Left: The density heatmap displaying the signature score of the response to type I IFN pathway in neutrophils. Right: The violin plot showing the signature score of the response to type I IFN pathway in the neCRSwNPs. (**K**) The dot plot depicting the gene expression level related to the response to type I IFN pathway in different neutrophil subsets. (**L**) The scatter plot showing the correlation between the signature score of the response to type I IFN pathway and the average *MX1* expression level in the scRNA-seq data of nasal polyps evaluated with Spearman’s rank test. (**M**) The scatter dot plot displaying the *MX1* mRNA level in control uncinate tissues and nasal polyps evaluated with the Kruskal-Wallis test with Dunn’s post hoc test (control, *n* = 13; neCRSwNP, *n* = 14; eCRSwNP, *n* = 14). *P < 0.05.

Recent studies have demonstrated that human neutrophils exist in distinct transcriptional states and exhibit heterogeneity (12, 13). We partitioned neutrophils into 10 clusters based on DEGs and calculated the ratio of observed to expected cell numbers (Ro/e) (Figure 3, A and B, and Supplemental Table 16). Cluster 9 was excluded from further analysis because it predominantly expressed the eosinophil marker CLC (Figure 3C) (17). Considering previous grouping strategies, 5 distinct transcriptional subsets of neutrophils were identified: S100A8^+^, GBP5^+^, CXCL8^+^, EPHB1^+^, and S100A12^+^ neutrophils (Figure 3D and Supplemental Table 17).

We assessed neutrophil tissue enrichment using Ro/e analysis (Figure 3, E and F). S100A8^+^, EPHB1^+^, and S100A12^+^ neutrophils were preferentially enriched among PB neutrophils, while CXCL8^+^ neutrophils were enriched in both neCRSwNP and eCRSwNP neutrophils. S100A8^+^ and S100A12^+^ neutrophils exhibited upregulation of S100 family members (S100A4, S100A6, and S100A8), MME, and LST1, which are associated with the maturation state of neutrophils (18). EPHB1^+^ neutrophils were more prevalent among PB neutrophils whose expression of upregulated genes (*IGF1R* and *EGR1*) increased in response to insulin stimulation. CXCL8^+^ neutrophils displayed upregulated expression of neutrophil activation-associated genes (*CXCL8*, *IL1B*, and *CD83*) and senescence-associated genes (*G0S2* and *CCL3L1*). In parallel, we observed greater neutrophil activation and senescence scores for the CXCL8^+^ subset (Figure 3, G–I). Pathway analysis revealed that the upregulated genes in CXCL8^+^ neutrophils were significantly associated with cytokine-mediated immune regulation. Therefore, CXCL8^+^ neutrophils represent activated states and play a proinflammatory role in CRSwNP.

We observed a higher proportion of GBP5^+^ neutrophils in neCRSwNP, which displayed elevated expression of IFN-inducible genes, including *ISG15*, *IFIT1*, *IFIT2*, *MX1*, and *GBP5* (Figure 3, F and G). Consistent with a recent report, GBP5^+^ neutrophils showed increased PD-L1 (*CD274*) expression with a potential immunosuppressive effect (11) (Supplemental Figure 3A). The response to IFN-I pathway (GO: 0034340) was enriched in GBP5^+^ neutrophils (Figure 3, J and K). Transcription factor analysis further showed increased IFN regulatory factor (*IRF*) and signal transducer and activator of transcription (*STAT*) in GBP5^+^ neutrophils (Supplemental Figure 3B and Supplemental Table 18). *MX1*, as an IFN-I–regulated signature gene (19), positively associated with the response to IFN-I pathway (*R* = 0.64, Figure 3L) and displayed significantly higher mRNA expression in neCRSwNP than normal controls (*P* < 0.05, Figure 3, L and M), indicating IFN-I pathway activity in neCRSwNP.

### Context-specific transcriptional features of neutrophil subsets in eCRSwNP and neCRSwNP.

**Figure 4:**
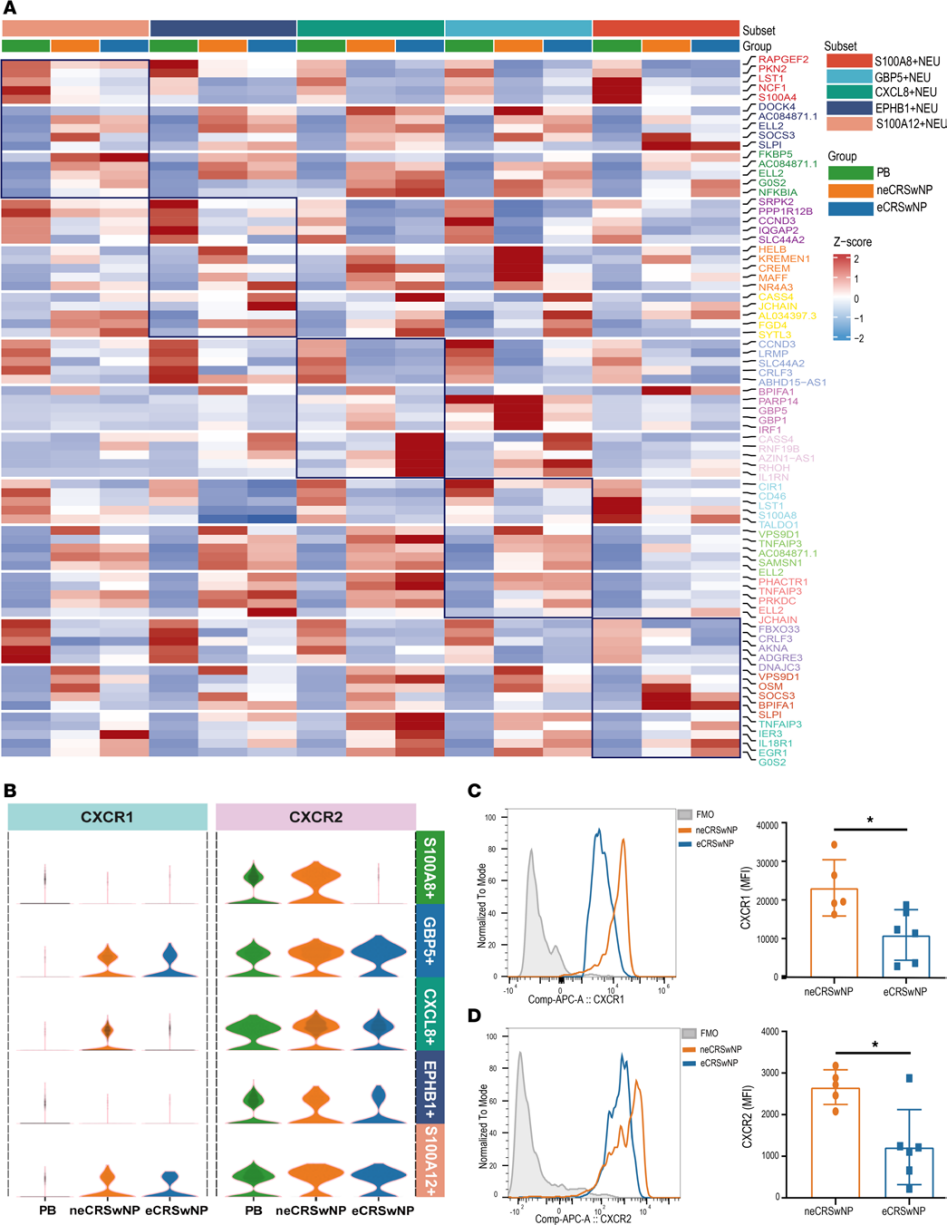
The transcriptional modulation of different neutrophil subsets in eCRSwNP and neCRSwNP. (**A**) Heatmap showing the top 5 gene expression levels for each group in different neutrophil subsets. (**B**) Violin plots showing the expression levels of C-X-C chemokine receptor type 1 (CXCR1) and CXCR2 in each neutrophil subset. (**C**) Representative flow cytometry histogram showing the levels of CXCR1. Graph shows the differences in the CXCR1 MFI ratio in nasal polyp neutrophils evaluated with the Mann-Whitney *U* test (neCRSwNP, *n* = 5; eCRSwNP, *n* = 6). (**D**) Representative flow cytometry histogram showing the levels of CXCR2 and graph showing the differences in the CXCR2 MFI ratio in nasal polyp neutrophils evaluated with the Mann-Whitney *U* test (neCRSwNP, *n* = 5; eCRSwNP, *n* = 6). *P < 0.05.

To further investigate the transcriptional differences in individual neutrophil subsets under distinct inflammatory backgrounds, we analyzed the DEGs in each subset (Figure 4A and Supplemental Tables 19–23). *BFIFA1*, encoding SPLUNC1, an antimicrobial protein, was mainly upregulated in the S100A8^+^, S100A12^+^, and CXCL8^+^ subsets in neCRSwNP. Additionally, CXCL8^+^ neutrophils from neCRSwNP patients exhibited upregulated expression of IFN-induced genes (*GBP5*, *GBP1*, and *IRF1*). S100A8^+^ and S100A12^+^ neutrophils in eCRSwNP patients overexpressed genes associated with neutrophil activation (*ERG1* and *TNFAIP3*).

Interestingly, we found that CXCL8 receptor (*CXCR1* and *CXCR2*) expression was notably lower in predominantly NP-infiltrating CXCL8^+^ neutrophils in eCRSwNP compared with neCRSwNP (Figure 4B). Flow cytometry confirmed decreased CXCR1 and CXCR2 expression on the eCRSwNP neutrophil surface (Figure 4, C and D, and Supplemental Figure 4A).

Overall, these results suggest that NP-infiltrating neutrophils exhibit heterogeneous functional states and are transcriptionally affected by different inflammatory patterns.

### OSM secreted by neutrophils is elevated in CRSwNP.

**Figure 5:**
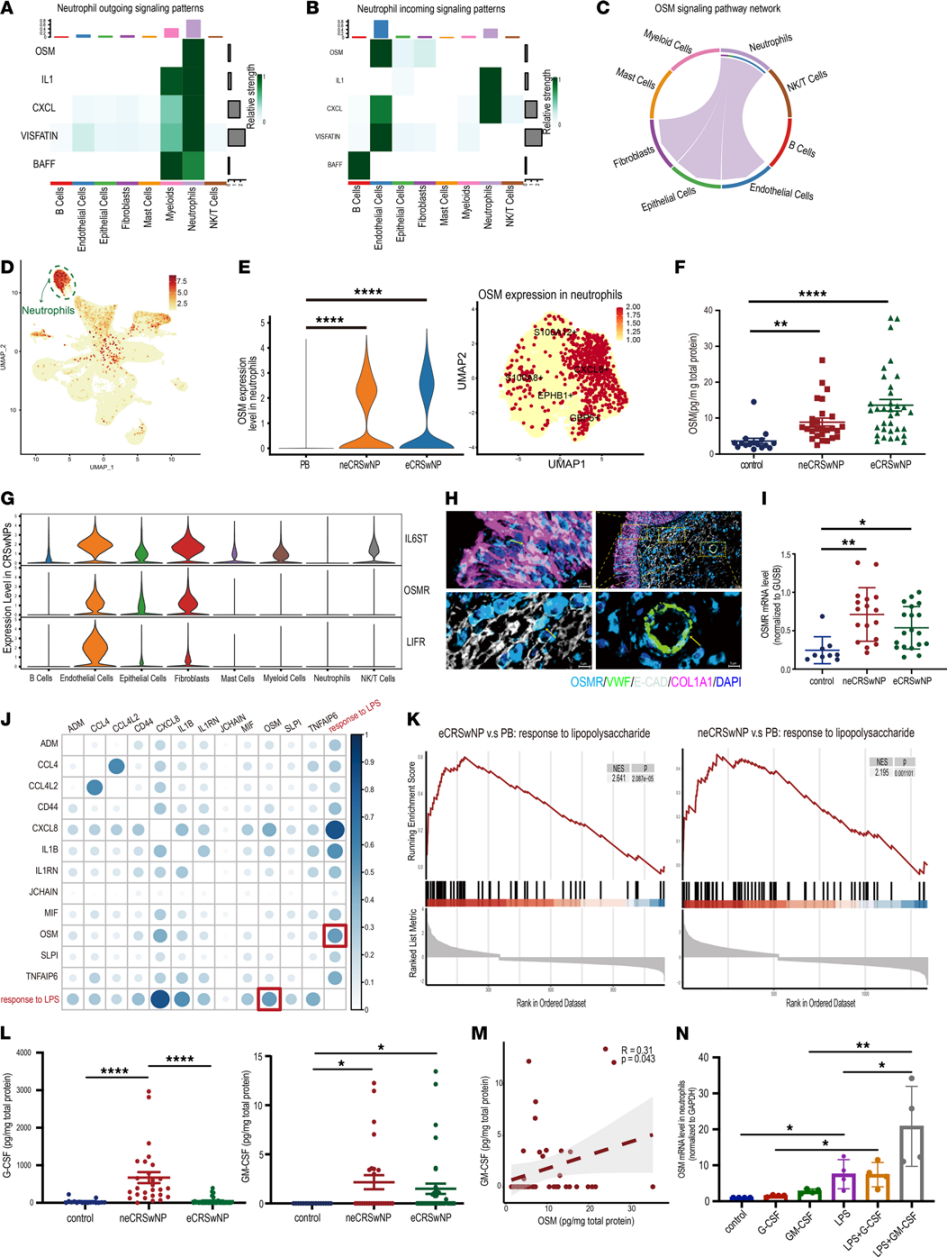
The level of OSM secreted by neutrophils is elevated in CRSwNP. (**A**) Heatmap showing the outgoing signaling patterns when neutrophils were selected as sender cells via the CellChat algorithm. (**B**) Heatmap showing the incoming signaling patterns when neutrophils were selected as sender cells via the CellChat algorithm. (**C**) Chord diagrams of the signaling pathway network displaying secreting and receiving cells of OSM signaling. (**D**) UMAP plot depicting the distribution of OSM in the scRNA-seq data. (**E**) Violin plot depicting the OSM expression level, and UMAP plot depicting the distribution of OSM in neutrophils of the scRNA-seq data. (**F**) Scatter dot plot depicting the protein levels of OSM in control uncinate tissue (UT) (*n* = 17), neCRSwNP (*n* = 27), and eCRSwNP (*n* = 34) groups in tissue homogenates evaluated with the Kruskal-Wallis test with Dunn’s post hoc test. (**G**) Violin plots showing the expression levels of the OSM receptors, including gp130 (*IL6ST*), LIFRα (*LIFT*), and OSMRβ (*OSMR*). (**H**) Representative images of OSMR, VWF (a biomarker of endothelial cells), E-CAD (a biomarker of epithelial cells), and COL1A1 (a biomarker of fibroblasts) immunofluorescent staining in nasal polyps. Scale bars: 5 μm. (**I**) Scatter dot plot displaying the *OSMR* mRNA level in control UTs and nasal polyps evaluated with the Kruskal-Wallis test with Dunn’s post hoc test (control, *n* = 9; neCRSwNP, *n* = 17; eCRSwNP, *n* = 19). (**J**) Heatmap presenting the correlation between the signature score of the response to LPS pathway and the expression level of common upregulated cytokines/chemokines in neCRSwNP and eCRSwNP neutrophils evaluated with Spearman’s rank test. (**K**) The GSEA plot showing the enrichment of response to LPS in the eCRSwNP or neCRSwNP neutrophils compared with the PB neutrophils. (**L**) The scatter dot plot depicting the protein levels of G-CSF and GM-CSF in control UT (*n* = 17), neCRSwNP (*n* = 27), and eCRSwNP (*n* = 34) tissue homogenates evaluated with the Kruskal-Wallis test with Dunn’s post hoc test. (**M**) The scatter plot visualizing the correlation of OSM and GM-CSF expression level in tissue homogenates of nasal polyps evaluated with Spearman’s rank test. (**N**) The histogram displaying *OSM* mRNA level in neutrophils after stimulation with LPS, G-CSF, and GM-CSF evaluated with the Kruskal-Wallis test with Dunn’s post hoc test (*n* = 4). *P < 0.05, **P < 0.01, ****P < 0.001.

To clarify the pathogenic role of neutrophils in both CRSwNP subendotypes, we further screened the DEGs between CRSwNP and PB neutrophils. Eighty-seven genes, including *OSM*, *CXCL8*, and *IL1B*, were uniformly upregulated in both neCRSwNP and eCRSwNP neutrophils (Supplemental Figure 5, A and B). Next, we analyzed biologically relevant communications between neutrophils and other cell types in NPs (Figure 5, A and B, and Supplemental Figure 5, C–F). Of particular interest was OSM, as the cell type–specific networks of OSM showed that neutrophils were the prominent source of effector secretion, with fibroblasts, epithelial cells, and endothelial cells being the primary targets (Figure 5C). The scRNA-seq data revealed neutrophils, especially CXCL8^+^ neutrophils, as the dominant cell type expressing OSM in NPs (Figure 5, D and E). Furthermore, ELISA analysis demonstrated higher OSM protein levels in NP homogenates from both neCRSwNP and eCRSwNP (Figure 5F).

There are 2 types of OSM receptors: type I (gp130/LIFRα complex) and type II (OSMRβ/gp130 complex). OSM receptors, including gp130 (*IL6ST*), LIFRα (*LIFT*), and OSMRβ (*OSMR*), are predominantly expressed in fibroblasts, epithelial cells, and endothelial cells (Figure 5G). A previous study showed that the OSMRβ/gp130 complex, not the gp130/LIFRα complex, is upregulated and mediates OSM responses in NPs (20). The localization of OSMR was confirmed through costaining of endothelial cells, epithelial cells, and fibroblasts with von Willebrand factor (VWF), E-cadherin (E-CAD), and collagen type I α1 chain (COL1A1), respectively (Figure 5H). Elevated OSMR expression was also detected in neCRSwNP and eCRSwNP (Figure 5I).

Inspired by recent findings that LPS promoted airway inflammation through OSM secretion from macrophages (21), we found a positive correlation between OSM expression and the LPS pathway response in NP-infiltrating neutrophils (Figure 5J). Functional enrichment further revealed a significantly higher response to LPS pathway in neCRSwNP or eCRSwNP neutrophils (Figure 5K).

GM-CSF and G-CSF, inducers of neutrophil differentiation and activation, exhibited elevated protein levels in neCRSwNP and eCRSwNP homogenates compared with those in control uncinate tissues (Figure 5L). GM-CSF also showed a significant positive correlation with OSM (*R* = 0.31, Figure 5M). To further assess the factors driving neutrophils to produce OSM, we treated PB neutrophils with LPS, G-CSF, and GM-CSF. Results suggested that LPS stimulated OSM expression and synergized with GM-CSF to enhance OSM production (Figure 5N).

### OSM modulates pathogenic pathways in epithelial cells and fibroblasts depending on inflammatory milieu.

**Figure 6:**
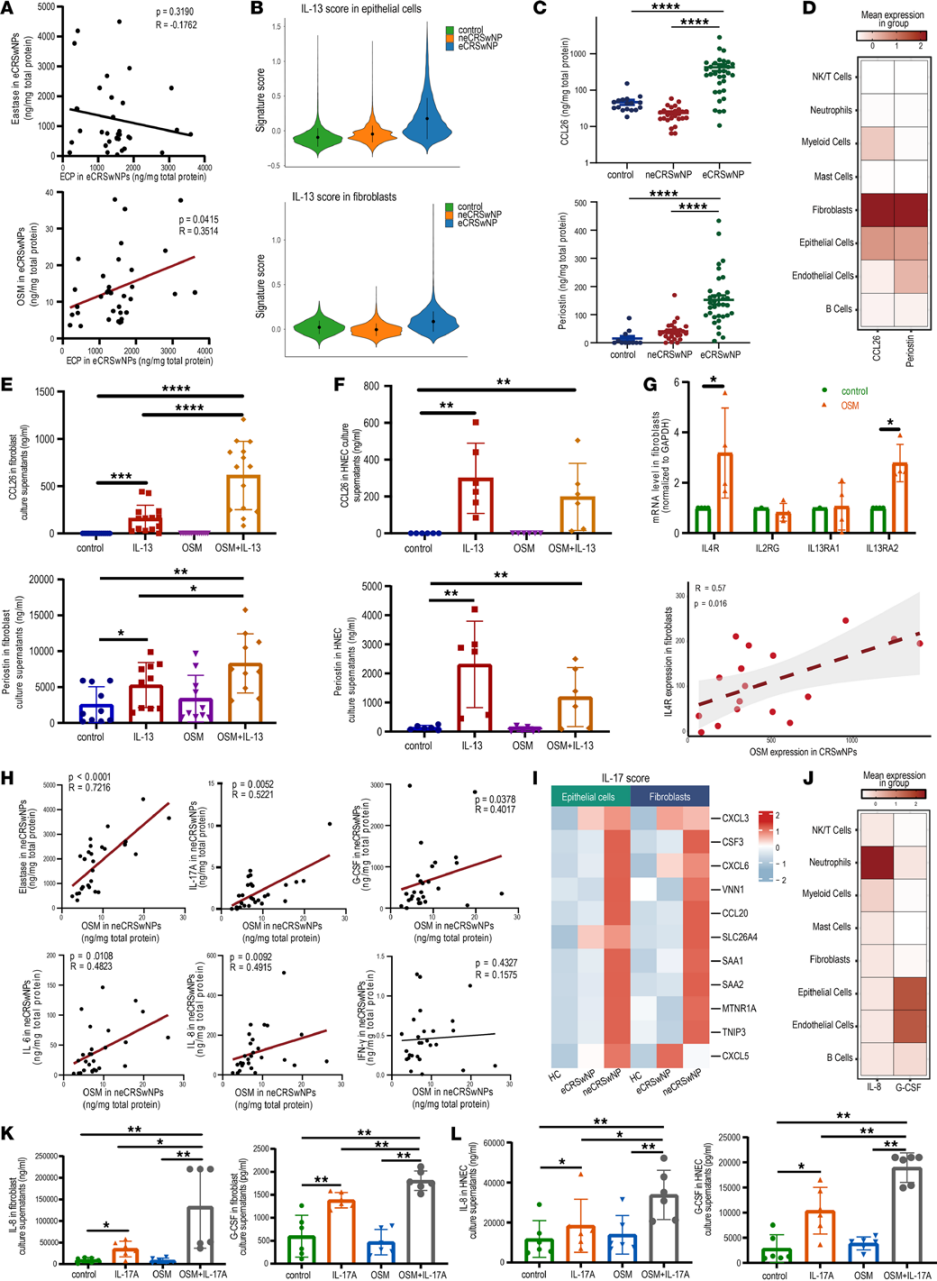
OSM modulates pathogenic pathways in epithelial cells and fibroblasts depending on inflammatory patterns. (**A**) The scatter plots showing the correlation of ECP and neutrophil elastase with OSM expression in eCRSwNP tissue homogenates. (**B**) The violin plots showing the signature score of the IL-13 pathway in fibroblasts and epithelial cells of the nasal mucosa. (**C**) The scatter dot plots depicting the protein levels of CCL26 and periostin in control uncinate tissue (UT) (*n* = 17), neCRSwNP (*n* = 27), and eCRSwNP (*n* = 34) tissue homogenates evaluated with the Kruskal-Wallis test with Dunn’s post hoc test. (**D**) The heatmap showing the expression levels of CCL26 and periostin in the scRNA-seq data. (**E**) The histograms displaying CCL26 and periostin secretion in culture supernatants after 24 hours of stimulation of fibroblasts. (**F**) The histograms displaying CCL26 and periostin secretion in culture supernatants after 24 hours of stimulation evaluated with the Kruskal-Wallis test with Dunn’s post hoc test (*n* = 6). (**G**) Top: The histogram displaying IL-13 receptor mRNA levels (*IL4R*, *IL2RG*, *IL13RA1*, and *IL13RA2*) in fibroblasts after 12 hours of stimulation with OSM evaluated with the Mann-Whitney *U* test (*n* = 4). Bottom: Scatter plot displaying the correlation between the *IL4R* expression level in fibroblasts and the total OSM expression level in the scRNA-seq data of nasal polyp samples evaluated with Spearman’s rank test. (**H**) Scatter plots showing the correlation between neutrophil elastase, IL-17A, G-CSF, IL-6, and IL-8 and the OSM expression level in neCRSwNP tissue homogenates evaluated with Spearman’s rank test. (**I**) Heatmap showing the IL-17 pathway score in fibroblasts and epithelial cells of healthy controls with normal ethmoid or sphenoid sinuses and nasal polyps in the scRNA-seq data. (**J**) Heatmap showing the expression levels of G-CSF and IL-8 in the scRNA-seq data. (**K**) Histograms displaying the G-CSF and IL-8 secretion in culture supernatants after 12 hours of stimulation in fibroblasts evaluated with the Kruskal-Wallis test with Dunn’s post hoc test (*n* = 6). (**L**) Histograms displaying the G-CSF and IL-8 secretion in culture supernatants after 12 hours of stimulation of HNECs evaluated with the Kruskal-Wallis test with Dunn’s post hoc test (*n* = 6). *P < 0.05; **P < 0.01; ***P < 0.001; ****P < 0.001.

Consistent with previous reports, NE expression did not significantly correlate with eosinophil marker (eosinophil cationic protein, ECP) expression in NP homogenates (6). However, we found that neutrophil-derived OSM expression was significantly positively correlated with ECP expression (*R* = 0.35, Figure 6A), which suggests the potential role of neutrophils in promoting eosinophilic inflammation in NPs. To further elucidate the OSM-regulated pathogenic pathway in eCRSwNP, we performed enrichment analysis of genes upregulated in OSM-targeted epithelial cells and fibroblasts. The scRNA-seq data revealed increased IL-13 signaling pathway scores in eCRSwNP epithelial cells and fibroblasts (Figure 6B). Additionally, downstream inflammatory factors of the IL-13 signaling pathway, such as CCL26 and periostin, were significantly increased in eCRSwNP polyp homogenates and mainly produced by fibroblasts and epithelial cells (Figure 6, C and D). We speculated that OSM may affect targeting cells by modulating the IL-13 immune response. Subsequently, we found that OSM significantly enhanced IL-13–dependent CCL26 and periostin production in fibroblasts, but not in epithelial cells (Figure 6, E and F). To explore the underlying mechanism, we examined IL-13 receptor distributions and changes following OSM stimulation. Interestingly, OSM significantly promoted *IL4R* and *IL13RA2* expression in fibroblasts (Figure 6G). The scRNA-seq data also showed a significant positive correlation between *IL4R* expression levels in fibroblasts and OSM expression levels in NPs (Figure 6G). These results suggest that neutrophils in eCRSwNP may amplify eosinophilic inflammation by regulating the IL-13–mediated immune response in fibroblasts.

Neutrophils are the main effector cells in neCRSwNP patients, and our previous work suggested prominent type 3 inflammation in our neCRSwNP cohort (22). We next sought to determine the regulatory role of OSM in neCRSwNP. We observed a positive correlation between OSM and NE, IL-17A, G-CSF, IL-6, and IL-8 in tissue homogenates (Figure 6H). A set of IL-17–responsive genes was commonly upregulated in epithelial cells, fibroblasts, and endothelial cells in neCRSwNP (Figure 6I). The pivotal downstream chemokines of IL-17, such as G-CSF, are predominantly expressed in epithelial cells. IL-8 was also expressed in epithelial cells and fibroblasts (Figure 6J). To investigate the modulatory effect of OSM on the type 3 inflammatory milieu, we utilized IL-17A in combination with OSM to stimulate fibroblasts and human nasal epithelial cells (HNECs). We found robust upregulation of G-CSF and IL-8 upon costimulation compared with IL-17A stimulation alone (Figure 6, K and L, and Supplemental Figure 6, E–H), suggesting neutrophils in neCRSwNP could intensify neutrophilic inflammation by releasing OSM, thereby enhancing the proinflammatory effect of IL-17A. However, this modulatory effect was not related to IL-17 receptors because their expression did not change after OSM stimulation (Supplemental Figure 6I). Nevertheless, OSM stimulation significantly upregulated NF-κB inhibitor-ζ (IκBζ) expression, a crucial mediator of IL-17A signaling in psoriasis (23), thereby possibly providing an additive effect on IL-17A’s effect in HNECs and fibroblasts (Supplemental Figure 6J).

As mentioned above, eCRSwNP and neCRSwNP patients exhibited consistently upregulated OSM expression and cellular sources from CXCL8^+^ neutrophils, suggesting that neutrophil-derived OSM specifically strengthens inflammation according to the tissue microenvironment.

## Discussion

It is well documented that infiltrated neutrophils serve as the main effector cells in CRSwNP patients with non–type 2 inflammation. The presence of neutrophils, as well as their activation status in type 2 CRSwNP, has also been observed in recent studies (6, 7, 24). However, comparisons of the functional status of neutrophils within different inflammatory backgrounds have not yet been performed. Our study is the first to our knowledge to utilize scRNA-seq to determine neutrophil functional diversity in NPs. We demonstrated activated neutrophils in both eCRSwNP and neCRSwNP based on scRNA-seq and CD62L flow cytometry. Given recent findings that neutrophils are highly heterogeneous, in contrast with the traditional view that neutrophils are a homogeneous antimicrobial cell population, we hypothesized and confirmed that NP-infiltrating neutrophils are composed of 5 functional subsets with distinct markers and that CXCL8^+^ and GBP5^+^ neutrophil subsets account for a greater proportion of NP-infiltrating neutrophils than blood neutrophils. We also provide the first evidence to our knowledge that transcriptomic modulation occurs in neutrophils after they migrate to NPs and that tissue-specific transcriptional regulation occurs in 2 CRSwNP endotypes. Furthermore, we found that the proinflammatory effect of CXCL8^+^ neutrophil–derived OSM on epithelial cells and fibroblasts was dependent on the tissue inflammatory microenvironment of CRSwNP.

Consistent with previous work, we observed a comparable and elevated number of infiltrated neutrophils in both eCRSwNP and neCRSwNP (24). We also confirmed highly activated neutrophils in eCRSwNP with lower CD62L expression, as detected by flow cytometry (6, 25). Although neutrophils have been conventionally considered as the main effector cells in neCRSwNP, we confirmed their activation status by decreasing the cell surface expression of CD62L. Recently, Poposki et al. performed bulk RNA-seq of sorted neutrophils from NPs and blood and confirmed the infiltrated neutrophil activation in NPs by GO analysis (6). In the present study, we analyzed neutrophils from NP tissue of both subtypes using scRNA-seq. Interestingly, the GO analysis of upregulated genes also demonstrated the activation of neutrophils in both CRSwNP subtypes. Moreover, we also observed a significant increase in *IL1B* expression in neutrophils from both CRSwNP subtypes. Inflammasome activation with increased IL-1β expression has been reported previously in eCRSwNP and neCRSwNP, associated with neutrophilic inflammation (26–28). Our scRNA-seq analysis indicated increased expression of inflammasome-related genes, such as *GSDMD*, *NLRP3*, and *AIM2*. Together, these findings indicate that activated neutrophils may contribute to the pathogenesis of both CRSwNP subtypes through activating inflammasome pathways.

The 5 established neutrophil subsets were conserved between PB and NPs, with CXCL8^+^ and GBP5^+^ neutrophils presenting higher activation and aging scores. Prior studies reported that tissue-infiltrating neutrophils aged more quickly and became more active (29, 30). Interestingly, the CXCL8^+^ neutrophils were more abundant in NPs from both subendotypes, confirming that the migration of neutrophils into NPs was transcriptionally activated. Particularly, the proportion of the GBP5^+^ neutrophils in neCRSwNP was significantly greater than that in eCRSwNP, and the CXCL8^+^ neutrophils in neCRSwNP patients also presented with elevated expression of IFN-induced genes. Moreover, elevated expression of MX1, which has been previously evaluated as a biomarker for predicting type I IFN activity, was observed in neCRSwNP (19). Taken together, these findings indicated the activation of type I IFN signaling in neCRSwNP. Traditionally, type I IFN activation is associated with protection from viral or bacterial infections and contributes to the perpetuation of inflammation in several autoimmune diseases (31–33). The molecular basis driving the activation of type I IFN in neutrophils from neCRSwNP, as well as its pathogenic role, remains to be elucidated.

Increased neutrophil infiltration is associated with steroid unresponsiveness in CRSwNP patients (34, 35). Elevated CCL4L2 expression in neutrophils is associated with inhaled corticosteroids in patients with asthma (36). Our present study revealed increased expression of CCL4L2 in eCRSwNP neutrophils, indicating the refractoriness of steroid treatment in patients with severe CRSwNP with mixed inflammation (Supplemental Figure 2A). Moreover, several neutrophil subsets from eCRSwNP displayed decreased expression of CXCR1 and CXCR2 in comparison with those from neCRSwNP. A recent study reported the downregulatory effect of IL-4 or IL-13 on CXCR1 and CXCR2, and the decreased expression of CXCR1 and CXCR2 in eCRSwNP was possibly related to eosinophilic inflammation (37). Neutrophil migration driven by CXCL8 depended on the surface abundance of CXCR1 and CXCR2. Therefore, obvious neutrophil infiltration in eCRSwNP may be induced by other chemotaxis factors (38). These data further demonstrated that neutrophils underwent transcriptome modulation in response to the distinct inflammatory milieu.

The IL-13–mediated immune response serves as a pivotal biological process in the pathogenesis of eCRSwNP, which has been confirmed by the excellent efficacy of dupilumab, which targets IL-4Rα (39). Epithelial cells and fibroblasts are the main effector cells of the IL-13–mediated immune response in eCRSwNP patients (40, 41). Cell communication analysis revealed functional interactions between neutrophils and multiple effector cells, including epithelial cells and fibroblasts (42). Moreover, the OSM-mediated signaling pathway was specific for neutrophil-mediated crosstalk with epithelial cells and fibroblasts. In line with a previous report, OSM was significantly upregulated in eCRSwNP and located in neutrophils (43). Our scRNA-seq analysis revealed that OSM was mainly produced by CXCL8^+^ neutrophils. Wang et al. reported that in combination with IL-4, OSM promotes HNECs to release thymic stromal lymphopoietin (TSLP) through upregulating IL-4Rα expression (44). Our work revealed that OSM synergizes with IL-13 to enhance the production of CCL26 and periostin by fibroblasts. Previous findings have demonstrated the critical role of CCL26 and periostin in regulating eosinophilic inflammation (45–47). These findings further support the concept that neutrophils could significantly amplify type 2 inflammation through releasing OSM.

Previous studies, including our recent report, have demonstrated that elevated IL-17A levels are associated with CRSwNP and neutrophilic inflammation (22). Additionally, using a murine CRSwNP model in which IL-17A is targeted indicates the pathogenic function of IL-17A (48, 49). Despite the equivalent numbers of infiltrated neutrophils and NE levels in eCRSwNP and neCRSwNP, we detected significantly greater IL-17A levels in neCRSwNP. This may imply a prominent pathogenic effect of IL-17A in neCRSwNP. Our study revealed that genes upregulated in multiple effector cells, including epithelial cells, fibroblasts, and endothelial cells, were enriched in the IL-17A–mediated immune response pathway, which further indicated the central role of IL-17A in the pathogenesis of neCRSwNP. OSM has been reported to activate STAT3 signaling in airway smooth muscle cells and enhance IL-6 and CCL2 expression synergistically with IL-17 (50, 51). We found equivalently elevated OSM expression in neCRSwNP and detected its communication with epithelial cells and fibroblasts, prompting further investigation into OSM in neCRSwNP. Our further in vitro experiments revealed the ability of OSM to amplify the proinflammatory effect of IL-17A on nasal epithelial cells and fibroblasts. A previous study revealed that IκBζ mediates the synergistic inflammatory response to IL-17 and TNF-α in fibroblasts (52). IκBζ expression can be induced through the transcription factor STAT3 or NF-κB (53–55). Collectively, our findings suggest that the synergistic effect of OSM may involve increased IκBζ expression mediated by STAT3 activation.

While our study provided what we believe is a novel understanding of the functional versatility and heterogeneity of NP neutrophils, it has several limitations. First, our cohort did not employ scRNA-seq to compare neutrophils from normal sinonasal tissue to NP neutrophils, as sufficient neutrophils from normal tissue could not be obtained. Second, we did not analyze circulating neutrophils from CRSwNP. Although recent bulk sequencing analysis did not reveal activation of CRSwNP PB neutrophils in comparison to control PB neutrophils, the phenotypic and transcriptomic features of PB neutrophils in CRSwNP deserve future clarification (6). Finally, we focused primarily on the transcriptomic features of NP neutrophils and complemented the analysis with flow cytometry and in vitro validation. Further studies combining mass cytometry and epigenomic approaches will fully define the phenotypic and functional features of NP-infiltrating neutrophils.

### Conclusions.

In conclusion, our study demonstrated that neutrophils are highly heterogeneous, with 5 functional subsets, and acquired transcriptional adaptation when exposed to an NP tissue environment. While neutrophils from both eCRSwNP and neCRSwNP have several overlapping functional features, we also observed context-specific transcriptional profiling. Furthermore, we revealed that neutrophils perform a modulatory role in the pathogenesis of CRSwNP by releasing OSM to interact with epithelial cells and fibroblasts and then amplifying eosinophilic or neutrophilic inflammation in a manner dependent on the inflammatory environment.

## Methods

### Sex as a biological variable.

Our study included both male and female patients.

### Clinical samples.

All participants, including CRSwNP patients and controls, were recruited from the Department of Otorhinolaryngology at the Eye and ENT Hospital of Fudan University. CRSwNP was diagnosed based on the criteria defined by recently released European and American guidelines (1, 2). Individuals with an isolated antrochoanal polyp, fungal rhinosinusitis, cystic fibrosis, or unilateral NP were excluded from the study. No participants used antibiotics or topical/oral corticosteroids for at least 1 month before the operation. The clinical characteristics of each patient, including age, sex, history of smoking, prior sinus surgery history, asthma status, and CT score, were collected and are listed in Supplemental Table 1. The Lund-Mackay staging score, maxillary sinus score, ethmoid sinus score, and E/M ratio (the ratio of the ethmoid and maxillary sinus scores) were assessed by an independent radiologist (56). eCRSwNP was defined as tissue eosinophils above or equal to 10 per high-power field (HPF) according to hematoxylin and eosin staining, whereas neCRSwNP was defined as the absence of evidence of eosinophilia (1, 22, 57). NP specimens were collected from CRSwNP patients who failed conservative medical therapy and underwent endoscopic sinus surgery. Patients who underwent endoscopic orbital decompression, cerebral spinal fluid leakage repair, or skull base surgery without a clinical or radiographic history of CRS, allergic rhinitis, or asthma were included as control individuals. Uncinate tissue was obtained from control individuals for subsequent biological analysis.

### scRNA-seq data.

The original FASTQ file data for NP tissues (5 neCRSwNP and 6 eCRSwNP samples) were retrieved from the GSA under accession number HRA000772 (57). Additionally, 5 PB samples from healthy controls were obtained from the GEO under accession number GSE157789 (58). These datasets were then integrated with our sequencing data from 6 NP tissues. In total, our analysis included 22 samples, consisting of 5 PB samples, 7 neCRSwNP samples, and 10 eCRSwNP samples (Figure 1A).

### Preparation of single-cell suspensions.

NP tissue samples (2 neCRSwNPs, 4 eCRSwNPs) were collected in MACS tissue storage solution (130-100-008, Miltenyi Biotec) within 30 minutes of the surgical procedure. Then, the samples were dissociated into single-cell suspensions by mechanical dissociation for 30 minutes with a gentleMACS Dissociator (130-093-235, Miltenyi Biotec), with 1 mg/mL collagenase I (Sigma-Aldrich) and 30 μg/mL DNase I (Sigma-Aldrich). The suspension was subsequently centrifuged at 300*g* for 5 minutes at 4°C, after which the single-cell suspension was filtered through a 40-μm nylon cell strainer (Falcon). Red blood cell lysis solution (Sigma-Aldrich) was further used to remove erythrocytes. A Dead Cell Removal Kit (Miltenyi Biotec) was utilized to remove dead cells, ensuring cell viability greater than 90%.

Following the manufacturer’s protocol, libraries were prepared for scRNA-seq using the Chromium Next GEM Single Cell 3′ Reagent Kit version 3.1 (10× Genomics). Briefly, single-cell suspensions were loaded onto a Chromium Single-Cell Controller Instrument (10× Genomics) to generate single-cell gel beads in emulsions (GEMs). After GEM generation, reverse transcription reactions were carried out to produce full-length barcode cDNA, followed by the disruption of emulsions using the recovery agent. Barcoded cDNA was subsequently purified with Dynabeads MyOne Silane beads (Thermo Fisher Scientific) and amplified by PCR with cycles adjusted based on the cell recovery rate. The amplified cDNA was then fragmented, end-repaired, A-tailed, index adapter–ligated, and used for library amplification. Library sequencing was performed on the Illumina sequencing platform (HiSeq X Ten), and 150-bp paired-end reads were generated. Then, we obtained the original FASTQ file data.

### scRNA-seq data preprocessing and quality control.

To process the data, we used the Cell Ranger software pipeline (version 6.1.2) provided by 10× Genomics. This pipeline allowed us to demultiplex cellular barcodes, map reads to the genome and transcriptome using the STAR aligner, and downsample reads as necessary to generate normalized aggregate data across samples. This process yielded a matrix of gene counts associated with individual cells.

To capture neutrophils in the raw data, we used the “cellranger count” command with the “force-cells” option to include low-UMI barcodes and the “include-introns” option to accommodate increased intron retention in neutrophils, as advised in the 10× Genomics official guide (59). We processed the unique molecular identifier (UMI) count matrix using the R package Seurat (version 4.0.3) (60). To accurately capture neutrophils while eliminating low-quality cells, a significant concern in microdroplet-based experiments, we applied the following criteria: (a) genes expressed in fewer than 3 cells were filtered out, (b) the number of detected genes was above 100, and (c) the percentage of mitochondrial genes was less than 50. To mitigate unexpected noise and expression artifacts, genes associated with mitochondria and ribosomes were excluded (Supplemental Figure 1A and Supplemental Table 2).

After applying these quality control criteria, the downstream analyses included 200,091 single cells with 31,215 genes. To obtain the normalized count, library size normalization was performed with the NormalizeData function in Seurat (61). Specifically, the datasets were normalized, multiplied by a scaling factor, and log-transformed using the LogNormalize function. After rescaling the integrated object, graph-based clustering was performed to cluster cells according to their gene expression profile using the FindClusters function, and uniform manifold approximation and projection (UMAP) and 2-dimensional *t*-distributed stochastic neighbor embedding (tSNE) calculations were performed (62). To merge samples and remove batch effects, we applied Harmony with default parameters to the first 30 principal components (PCs) to obtain the corrected PC embeddings (63). Then, UMAP and tSNE were generated again based on the Harmony Reduction (Supplemental Figure 1B) (63). Clustering (resolution: 0.3) was determined by evaluating cluster stability using the Clustree package (64). Finally, cell types were identified based on prior articles and reference transcriptomic datasets, such as the Human Primary Cell Atlas (Supplemental Table 3 and Supplemental Figure 1C) (22, 65, 66).

### Analytical strategies for neutrophils.

The subset of neutrophils was selected for further analysis. Harmony and Seurat were used for removing batch effects, dimension reduction, clustering, and differential gene expression. A resolution parameter of 0.8 was used for clustering. To annotate cell clusters, the DEGs for each cell cluster were identified by comparing each cluster to all other clusters using the FindAllMarkers function.

### Tissue distribution of clusters.

We compared the ratio of observed to expected cell numbers (Ro/e) in different tissues to quantify the tissue preference of each cluster using the epitools R package (67). One cluster was identified as enriched in a specific tissue if Ro/e was greater than 1.

### Pseudotime analysis.

We determined the developmental pseudotime of neutrophils with the Monocle2 package (68). Initially, the data matrix was converted from the Seurat object to the CellDataSet object using the new CellDataSet function. We used the differentialGeneTest function package to select ordering genes with a *q* value of less than 0.01, which is likely to be informative for ordering cells along the pseudotime trajectory. Dimensional reduction clustering analysis was performed with the reduceDimension function, followed by trajectory inference with the orderCells function. The top 60 genes that changed as a function of pseudotime were identified and visualized using the plot_pseudotime_heatmap function.

### DEGs and PPI analysis.

DEGs were identified using Seurat’s FindMarkers function and the MAST test. The criteria for significance were set at a *P* value of less than 0.05 and an absolute fold change (FC) of 2 or greater. Volcano plots were generated using the EnhancedVolcano package. For an in-depth exploration of the interactions between these DEGs, we conducted a PPI analysis based on the STRING database (69). To further identify subnetworks of DEGs, we utilized the MCODE plugin to screen modules of the PPI network. The results were visualized with Cytoscape (version 3.10.0) (70).

### Functional enrichment and gene set enrichment analysis.

GO enrichment, Kyoto Encyclopedia of Genes and Genomes (KEGG) pathway enrichment, and gene set enrichment analysis (GSEA) of DEGs were performed using the clusterProfiler package (71). The results were visualized by the clusterProfiler and GseaVis packages. Next, we calculated the enrichment of multiple genes across different cell clusters and calculated the gene set signature scores utilizing Seurat’s AddModuleScore function and the irGSEA package. We obtained gene sets characterizing the response to type I IFN (GO: 0034340) and neutrophil activation (GO: 0042119) from the GO database. We predicted senescence-associated pathways through published gene sets (72).

### Transcription factor analysis.

Transcription factor activity was calculated using the VIPER (version 1.32.0) and DoRothEA (version 1.10.0) packages (73, 74). The transcription factor activity was calculated separately for individual cells within each section using regulons with confidence intervals A, B, and C. Then, the estimated enrichment score was calculated based on the *z* score of the DEGs and was normalized for hierarchical clustering.

### Cellular crosstalk.

To assess the cellular crosstalk in NP tissues, we divided the subsets of the neCRSwNP and eCRSwNP groups for further analysis. Following dimension reduction, clustering, and cell type annotation, we quantified cellular crosstalk with the CellChat package (version 1.5.0) based on the curated ligand-receptor interaction database known as CellChatDB (42). In brief, the data matrix of NP tissues was converted from the Seurat object to a CellChat object using the createCellChat function. The total numbers of interactions and interaction strengths were computed using the computeCommunProb function, and the communication probabilities for each cell signaling pathway were calculated using the computeCommunProbPathway function.

### IL-13 and IL-17 signature score analysis in CRS patients.

The IL-13/IL-17 signature score was obtained using IL-13/IL-17 pathway–related genes as previously reported and validated (75). We integrated the scRNA-seq data of control mucosa from normal ethmoid or sphenoid sinuses (HRA000772) and NP tissues from CRSwNP patients (57). The dataset consisted of 5 healthy controls, 7 neCRSwNP samples, and 10 eCRSwNP samples. The entire data processing pipeline, quality control, dimension reduction, clustering, and cell type annotation were assessed as described above (data not shown). Heatmaps were visualized with the ComplexHeatmap R package (76).

### Tissue immunohistochemistry.

Tissue immunohistochemical staining was performed as previously described (77). Immunohistochemical staining of NE with a monoclonal antibody (1:800; ab131260, Abcam) was applied to assess neutrophil infiltration. Sections were evaluated by 2 independent observers who were blinded to the groups and treatments.

### Tissue immunofluorescence.

Tissue immunofluorescent staining was also performed as previously described (22). To determine the localization of OSMR, immunofluorescent staining was conducted with anti-OSMR (1:200; 10982-1-AP, Proteintech), DAPI for nuclei, anti-VWF (1:200; ab6994, Abcam) for endothelial cells, anti–E-CAD (1:400; 3195s, Cell Signaling Technology) for epithelial cells, and anti-COL1A1 (1:400; 72026s, Cell Signaling Technology) for fibroblasts.

### Quantitative real-time reverse transcription PCR and cytokine measurement.

Total RNA was extracted from tissue and cell samples using an RNA Easy Fast Tissue/Cell Kit (DP451, Tiangen Biotech) and then reverse transcribed to cDNA with a PrimeScript RT Master Mix Kit (RR036, TaKaRa). Quantitative real-time (RT) reverse transcription PCR was conducted using SYBR premix (RR820, Takara) with appropriate primers. Specific primers and TaqMan probes (Supplemental Table 4) were used to perform the quantitative RT-PCR amplification reactions.

The protein levels of ECP (7618E, MBL), G-CSF (EK0360, Boster), IL-8 (EHC008.96, Neobioscience), periostin (EK0985, Boster), and CCL26 (DY347, R&D Systems) in the cell culture supernatants were detected by using commercial ELISA kits according to the manufacturer’s instructions. OSM, IFN-γ, IL-5, IL-13, GM-CSF, and IL-17A were analyzed using a custom Human Cytokine/Chemokine Panel II Kit (Millipore).

### Isolation and in vitro cell stimulation.

As previously described (22), neutrophils were isolated from the PB samples of healthy volunteers via Ficoll-Hypaque gradient centrifugation and CD16 microbeads (130-045-701, Miltenyi Biotec). The purified neutrophils were stimulated with G-CSF (25 ng/mL; 300-23, Peprotech), GM-CSF (25 ng/mL; 300-03, Peprotech), and LPS (10 μg/mL; tlrl-eblps, Invitrogen) in RPMI 1640 supplemented with 10% FBS for 4 hours, after which the cells were harvested for further analysis.

HNECs were cultured in bronchial epithelial cell growth media (BEGM), and fibroblasts were cultured in RPMI 1640 supplemented with 10% FBS (22). HNECs and fibroblasts were stimulated with OSM (10 ng/mL; 8475-OM, R&D Systems), IL-17A (10 ng/mL; 317-ILB-050, R&D Systems), or IL-13 (10 ng/mL; 213-ILB-010, R&D Systems) for 12 or 24 hours. Cultured cells and their supernatants were collected for further analysis.

### Flow cytometric analysis of PB and NP tissue.

PB samples were collected from patients before surgery. NP tissues were collected within 30 minutes of the surgical procedure. The preparation of single-cell suspensions from both PB and NP samples followed the same procedure outlined earlier for scRNA-seq suspension preparation. Cell surface staining was performed for 30 minutes at 4°C with the following human fluorochrome-conjugated antibodies: anti-CD45, -CD66B, -CD16, -CD62L, -CXCR1, and -CXCR2. The primary antibodies used are listed in Supplemental Table 5. The stained cells were analyzed immediately on a FACSCelesta cytometer (BD Biosciences) using FlowJo (version 10.0).

### Statistics.

All data are presented as the mean ± SD and were analyzed using R (version 4.1.2), GraphPad Prism (version 8.0), and SPSS (version 23.0, IBM Corporation). Group differences were analyzed by 1-way ANOVA, the Kruskal-Wallis test with Dunn’s multiple-comparison test, or the Mann-Whitney *U* test. Correlations were analyzed by Spearman’s rank test. A *P* value of less than 0.05 was considered to indicate statistical significance with Bonferroni’s correction for multiple comparisons.

### Study approval.

All participants signed an informed consent form, and the study was approved by the Ethics Committee of the Eye and ENT Hospital of Fudan University.

### Data availability.

Raw sequencing data reported in this paper have been deposited at the GSA under HRA006614. Other data that support the findings of this study are available in the Supporting Data Values file or from the corresponding authors upon reasonable request.

## Author contributions

XS, HW, LH, and DW conceived, supervised, and supported study. HW, CZ, and QZ established the methodology of nasal tissue dissection, processing, and cell isolation. CZ, QZ, and JC performed tissue dissection, immunostaining analysis, and in vitro studies. FC, YW, YG, Y Zhou, and YY performed procurement of nasal tissue and analysis of immunostaining data. CZ analyzed the scRNA-seq data. HL, LS, KX, HY, and DW provided nasal tissue samples for cell culture. XS, HW, LH, JL, and DW assisted with clinical expertise and resources. Y Zang, HW, and XS performed integrated data analysis and interpretation of data. CZ, QZ, and HW wrote the manuscript. All authors read and approved the final version of the manuscript, take responsibility for its content, and agreed to submission.

## Supplementary Material

Supplemental data

Supplemental tables 1-23

Supporting data values

## Version 1 10/03/2024

In-Press Preview

## Version 2 11/22/2024

Electronic publication
