# Supplementary material for: Neutrophils in nasal polyps exhibit transcriptional adaptation and proinflammatory roles that depend on local polyp milieu
Source: JCI Insight. 2024 Nov 22;9(22):e184739. doi: 10.1172/jci.insight.184739 (PMC11601912; doi:10.1172/jci.insight.184739)
Supplement: Supplemental data [file jciinsight-9-184739-s221.pdf]

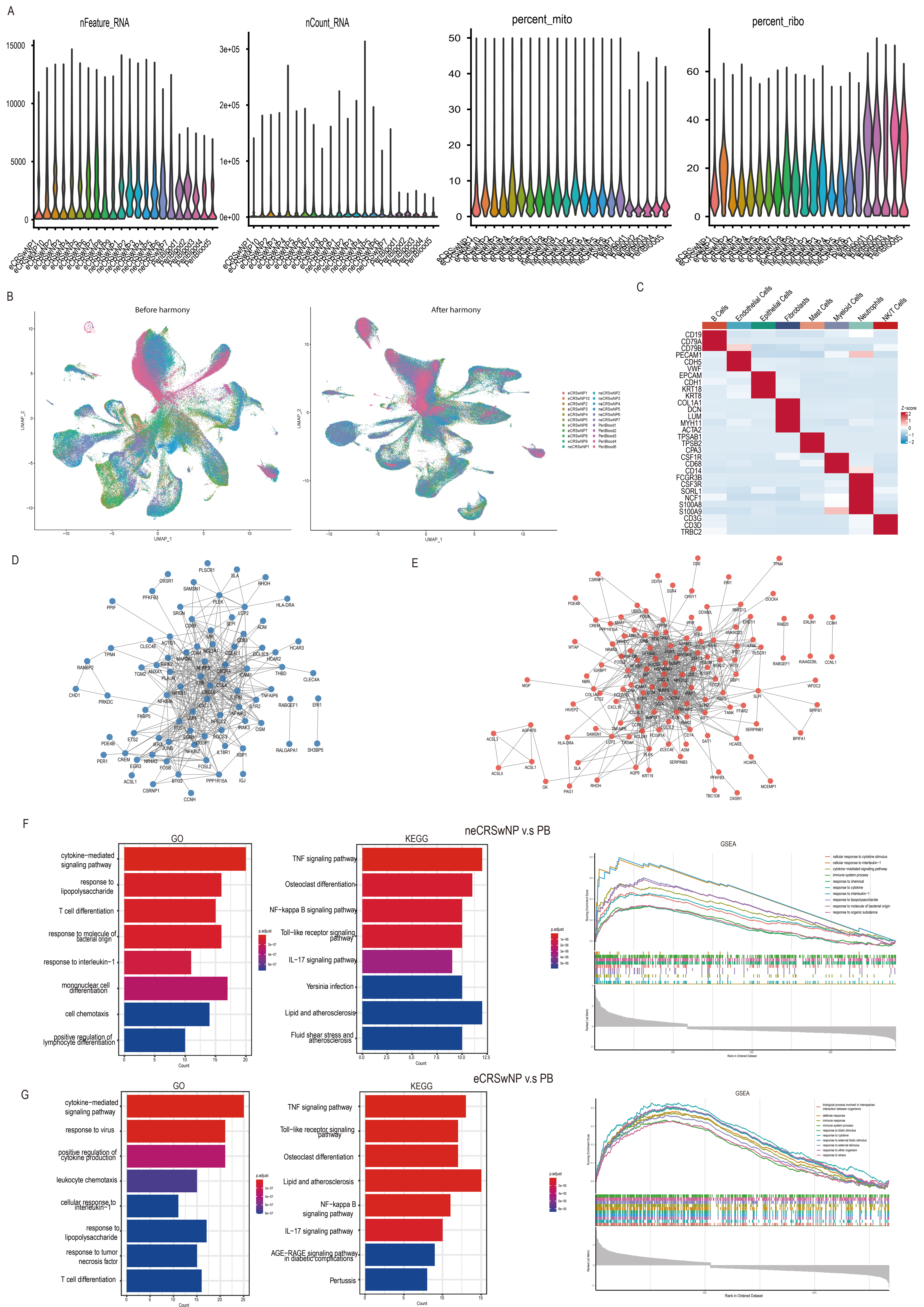
A
nFeature_RNA
nCount_RNA
percent_mito
percent_ribo
B
Before harmony
After harmony
UMAP_1
UMAP_2
C
B Cells
Endothelial Cells
Epithelial Cells
Fibroblasts
Mast Cells
Myeloid Cells
Neutrophils
NK/T Cells
CD19
CD79A
CD79B
PECAM1
CDH5
VWF
EPCAM
CDH1
KRT18
KRT8
COL1A1
DCN
LUM
MYH11
ACTA2
TPSAB1
TPSB2
CPA3
CSF1R
CD68
CD14
FCGR3B
CSF3R
SORL1
NCF1
S100A8
S100A9
CD3G
CD3D
TRBC2
D
E
F
neCRSwNP v.s PB
GO
cytokine-mediated signaling pathway
response to lipopolysaccharide
T cell differentiation
response to molecule of bacterial origin
response to interleukin−1
mononuclear cell differentiation
cell chemotaxis
positive regulation of lymphocyte differentiation
Count
p.adjust
KEGG
TNF signaling pathway
Osteoclast differentiation
NF-kappa B signaling pathway
Toll−like receptor signaling pathway
IL−17 signaling pathway
Yersinia infection
Lipid and atherosclerosis
Fluid shear stress and atherosclerosis
GSEA
G
eCRSwNP v.s PB
GO
cytokine−mediated signaling pathway
response to virus
positive regulation of cytokine production
leukocyte chemotaxis
cellular response to interleukin−1
response to lipopolysaccharide
response to tumor necrosis factor
T cell differentiation
KEGG
TNF signaling pathway
Toll−like receptor signaling pathway
Osteoclast differentiation
Lipid and atherosclerosis
NF−kappa B signaling pathway
IL−17 signaling pathway
AGE−RAGE signaling pathway in diabetic complications
Pertussis
GSEA

A

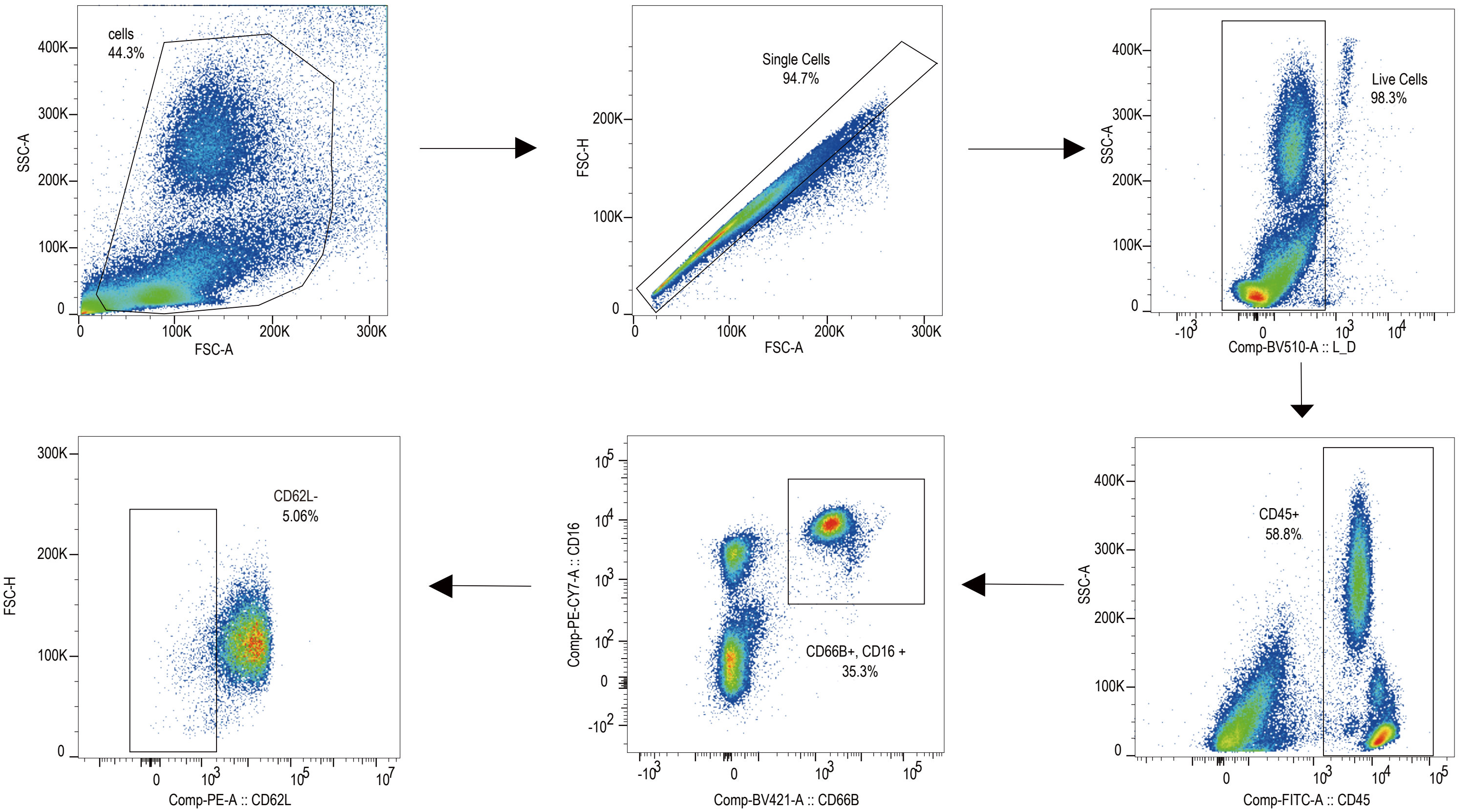

cells
44.3%
SSC-A
FSC-A
Single Cells
94.7%
FSC-H
FSC-A
Live Cells
98.3%
SSC-A
Comp-BV510-A :: L_D
CD45+
58.8%
SSC-A
Comp-FITC-A :: CD45
CD66B+, CD16 +
35.3%
Comp-PE-CY7-A :: CD16
Comp-BV421-A :: CD66B
CD62L-
5.06%
FSC-H
Comp-PE-A :: CD62L

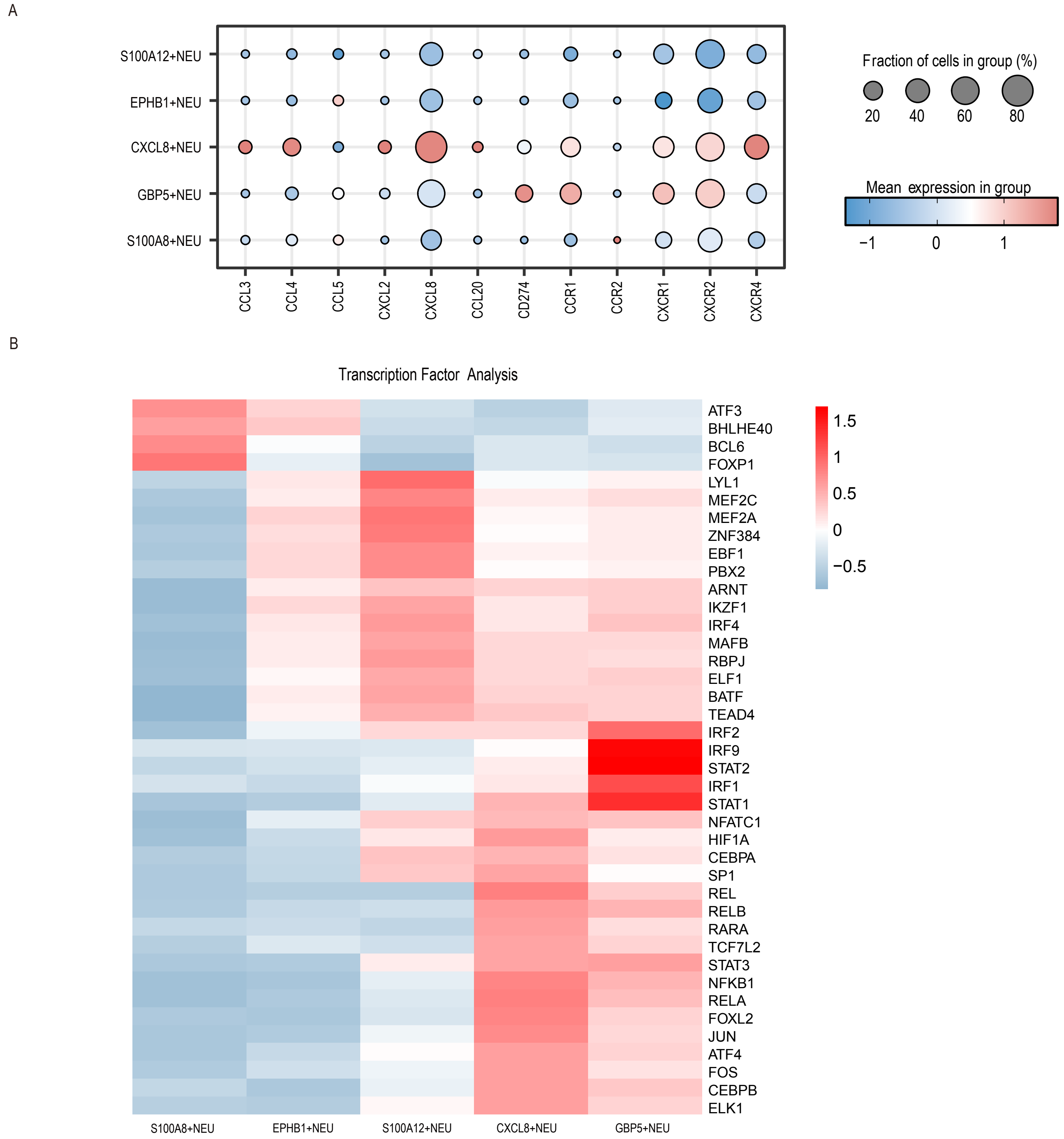
A
S100A12+NEU
EPHB1+NEU
CXCL8+NEU
GBP5+NEU
S100A8+NEU
CCL3
CCL4
CCL5
CXCL2
CXCL8
CCL20
CD274
CCR1
CCR2
CXCR1
CXCR2
CXCR4
Fraction of cells in group (%)
20
40
60
80
Mean expression in group
−1
0
1
B
Transcription Factor Analysis
ATF3
BHLHE40
BCL6
FOXP1
LYL1
MEF2C
MEF2A
ZNF384
EBF1
PBX2
ARNT
IKZF1
IRF4
MAFB
RBPJ
ELF1
BATF
TEAD4
IRF2
IRF9
STAT2
IRF1
STAT1
NFATC1
HIF1A
CEBPA
SP1
REL
RELB
RARA
TCF7L2
STAT3
NFKB1
RELA
FOXL2
JUN
ATF4
FOS
CEBPB
ELK1
1.5
1
0.5
0
−0.5
S100A8+NEU
EPHB1+NEU
S100A12+NEU
CXCL8+NEU
GBP5+NEU

A

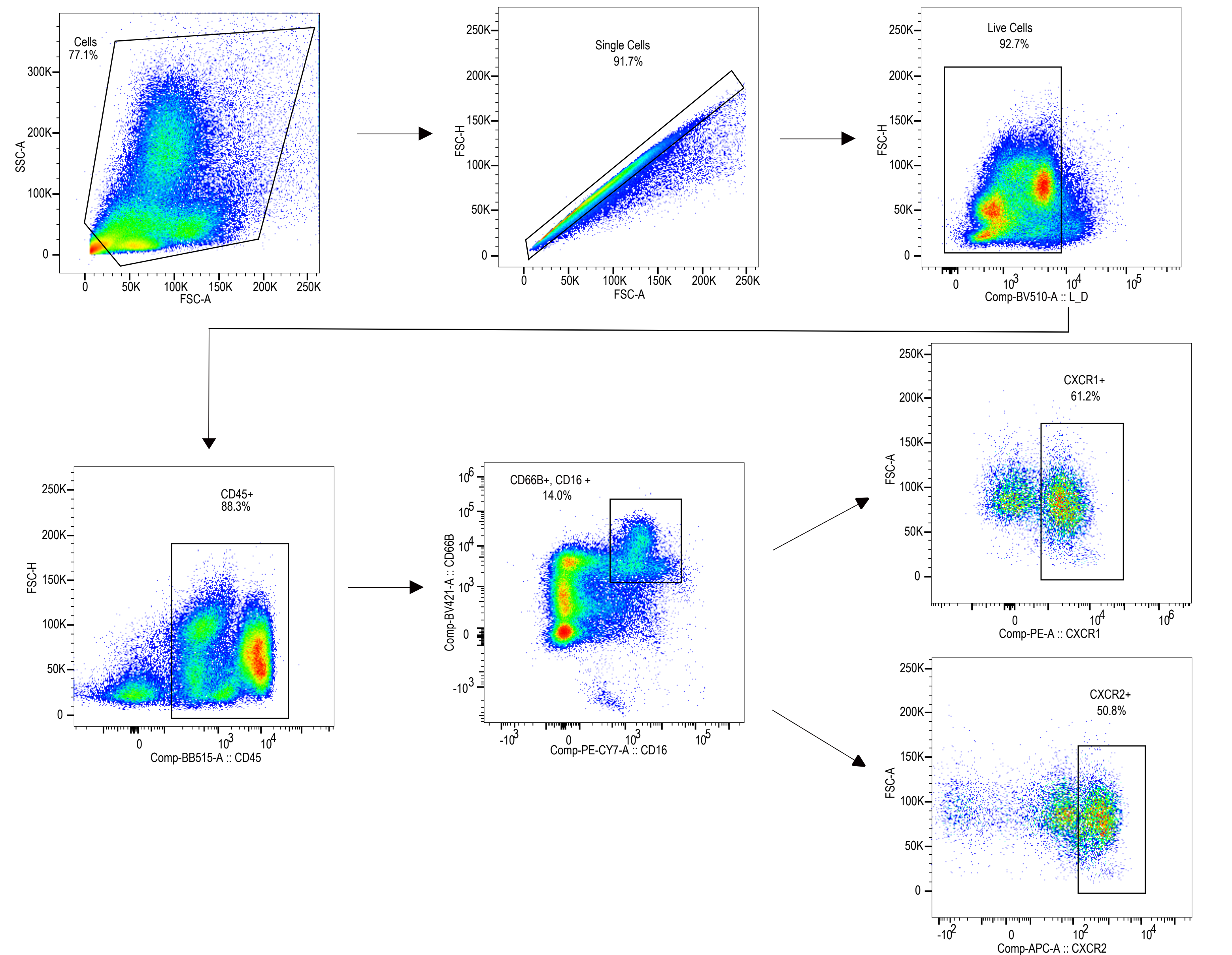

Cells
77.1%
SSC-A
FSC-A
Single Cells
91.7%
FSC-H
FSC-A
Live Cells
92.7%
FSC-H
Comp-BV510-A :: L_D
CD45+
88.3%
FSC-H
Comp-BB515-A :: CD45
CD66B+, CD16 +
14.0%
Comp-BV421-A :: CD66B
Comp-PE-CY7-A :: CD16
CXCR1+
61.2%
FSC-A
Comp-PE-A :: CXCR1
CXCR2+
50.8%
FSC-A
Comp-APC-A :: CXCR2

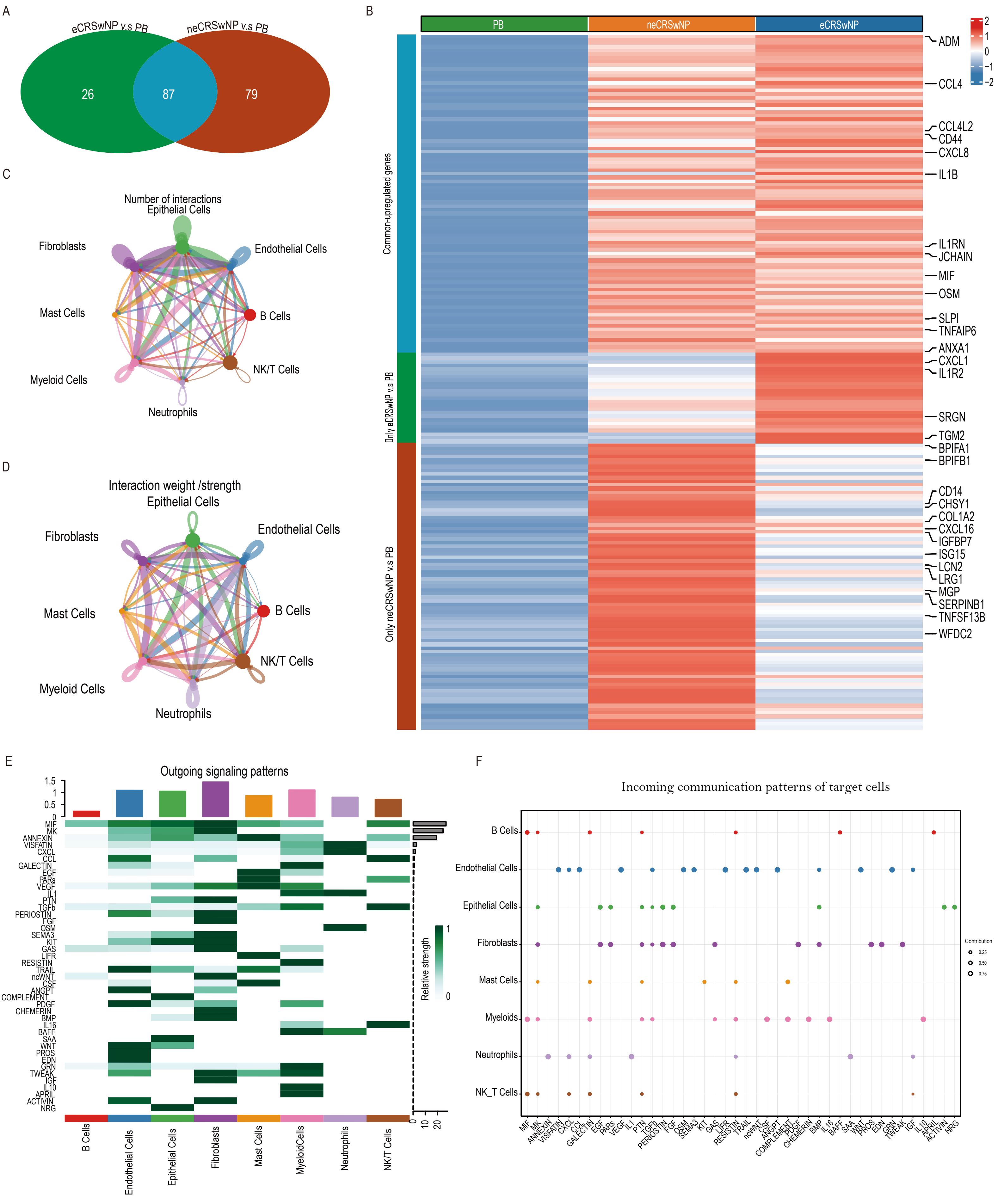
A
eCRSwNP v.s PB
neCRSwNP v.s PB
26
87
79
B
PB
neCRSwNP
eCRSwNP
Common-upregulated genes
Only eCRSwNP v.s PB
Only neCRSwNP v.s PB
ADM
CCL4
CCL4L2
CD44
CXCL8
IL1B
IL1RN
JCHAIN
MIF
OSM
SLPI
TNFAIP6
ANXA1
CXCL1
IL1R2
SRGN
TGM2
BPIFA1
BPIFB1
CD14
CHSY1
COL1A2
CXCL16
IGFBP7
ISG15
LCN2
LRG1
MGP
SERPINB1
TNFSF13B
WFDC2
C
Number of interactions
Epithelial Cells
Fibroblasts
Endothelial Cells
Mast Cells
B Cells
Myeloid Cells
NK/T Cells
Neutrophils
D
Interaction weight /strength
E
Outgoing signaling patterns
Relative strength
B Cells
Endothelial Cells
Epithelial Cells
Fibroblasts
Mast Cells
MyeloidCells
Neutrophils
NK/T Cells
F
Incoming communication patterns of target cells
Myeloids
NK_T Cells
Contribution
MIF MK ANNEXIN VISFATIN CXCL CCL GALECTIN EGF PARs VEGF IL1 PTN TGFb PERIOSTIN FGF OSM SEMA3 KIT GAS LIFR RESISTIN TRAIL ncWNT CSF ANGPT COMPLEMENT PDGF CHEMERIN BMP IL16 BAFF SAA WNT PROS EDN GRN TWEAK IGF IL10 APRIL ACTIVIN NRG

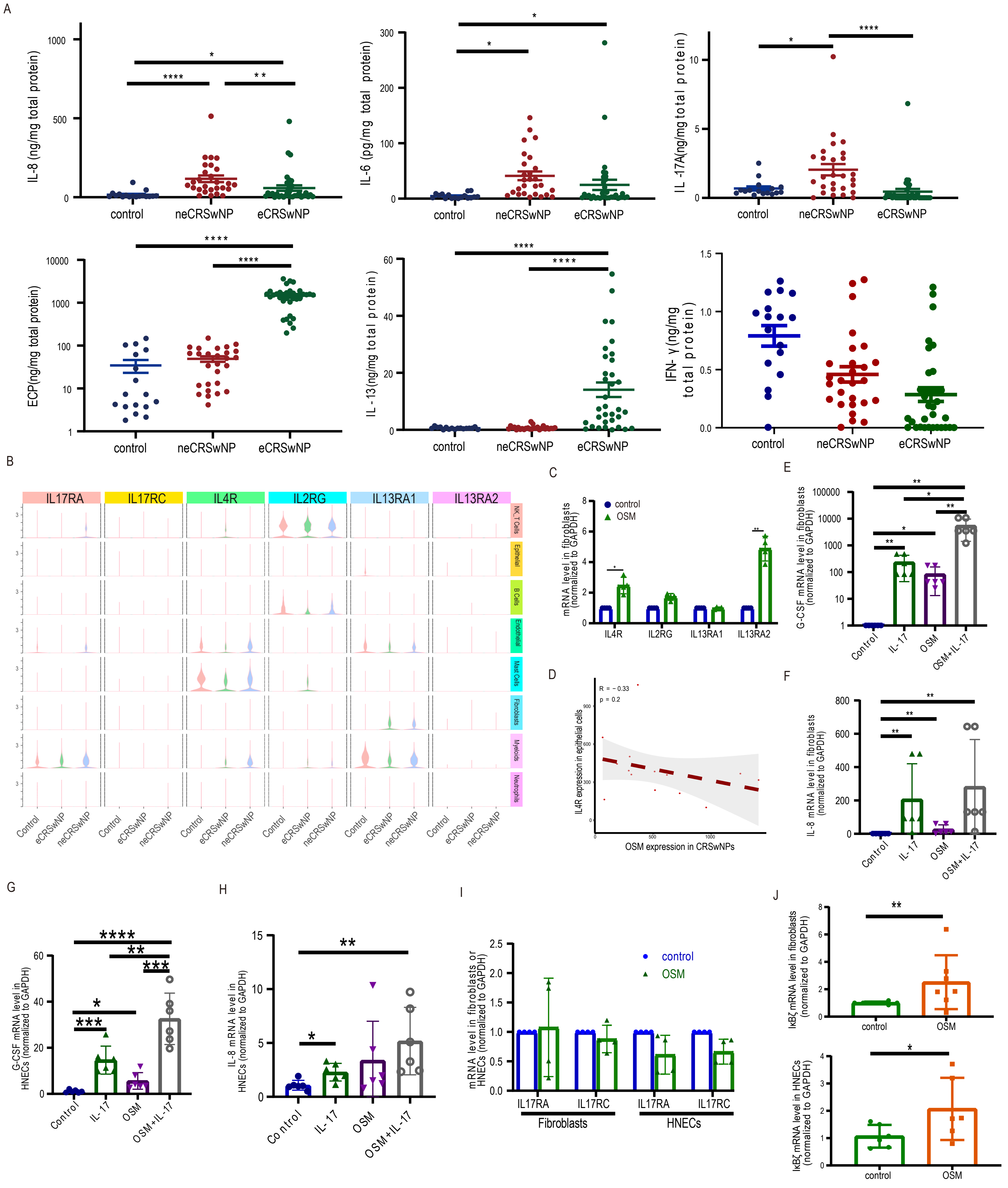
A
B
C
D
E
F
G
H
I
J
IL-8 (ng/mg total protein)
IL-6 (pg/mg total protein)
IL-17A(ng/mg total protein)
ECP(ng/mg total protein)
IL-13(ng/mg total protein)
IFN-γ (ng/mg total protein)
control
neCRSwNP
eCRSwNP
IL17RA
IL17RC
IL4R
IL2RG
IL13RA1
IL13RA2
NK_T Cells
Epithelial
B Cells
Endothelial
Mast Cells
Fibroblasts
Myeloids
Neutrophils
Control
mRNA level in fibroblasts (normalized to GAPDH)
OSM
IL2RG
IL13RA2
G-CSF mRNA level in fibroblasts (normalized to GAPDH)
IL-17
OSM+IL-17
IL4R expression in epithelial cells
OSM expression in CRSwNPs
R = −0.33
p = 0.2
IL-8 mRNA level in fibroblasts (normalized to GAPDH)
G-CSF mRNA level in HNECs (normalized to GAPDH)
IL-8 mRNA level in HNECs (normalized to GAPDH)
mRNA level in fibroblasts or HNECs (normalized to GAPDH)
Fibroblasts
HNECs
IκBζ mRNA level in fibroblasts (normalized to GAPDH)
IκBζ mRNA level in HNECs (normalized to GAPDH)

Supplemental material

Supplemental Figure Legends

Supplemental Figure 1. (A) Violin plots showing the number of genes and counts and the percentage of mitochondrial and ribosomal genes. (B) UMAP plot displaying the distribution of the samples before and after removing the batch effects. (C) Heatmap visualizing marker gene expression of each cell type. (D) Core network showing the protein–protein interaction (PPI) network for upregulated genes in the neCRSwNP neutrophils compared to the PB neutrophils. The PPI network of significantly upregulated genes (p value < 0.05 and FC ≥ 2) was constructed utilizing the STRING database, including 123 nodes and 598 edges. (E) The core network showing the PPI network for genes upregulated in eCRSwNP neutrophils compared to PB neutrophils. The PPI network of significantly upregulated genes (p value < 0.05 and FC ≥ 2) was constructed utilizing the STRING database and included 80 nodes and 384 edges. (F) The bar and GSEA plots showing the results of GO, KEGG, and GSEA pathway enrichment in the neCRSwNP neutrophils compared to the PB neutrophils. (G) The bar plots showing the results of GO, KEGG, and GSEA pathway enrichment in the eCRSwNP neutrophils compared to the PB neutrophils.

Supplemental Figure 2. (A) A representative cell sorting strategy showing activated neutrophils within the live CD45+CD66B+CD16+CD62L- population.

Supplemental Figure 3. (A) Dot plot depicting the gene expression levels of chemokines and chemokine receptors in different neutrophil subsets. (B) Heatmap

visualizing transcription factor analysis of DEGs in different neutrophil subsets.

Supplemental Figure 4. (A) A representative cell sorting strategy showing CXCR1+ or CXCR2+ neutrophils within the live CD45+CD66B+CD16+ population.

Supplemental Figure 5. (A) Venn diagram showing upregulated genes in the neCRSwNP and eCRSwNP neutrophils compared to the PB neutrophils. (B) Heatmap showing 87 common upregulated genes (including 12 cytokines/chemokines) in the neCRSwNP and eCRSwNP neutrophils. (C) Network graphs showing the number of CellChat interactions. (D) Network graphs showing the CellChat interaction weights. (E) Heatmap visualizing outgoing signaling patterns of CellChat. (F) Heatmap visualizing incoming communication patterns of CellChat target cells.

Supplemental Figure 6. (A) Scatter dot plot depicting the protein levels of ECP, IL-13, IL-8, and G-CSF in tissue homogenates from the control UT (n = 17), neCRSwNP (n = 27), and eCRSwNP (n = 34) groups. (B) Violin plots showing the expression levels of IL-13 receptors and IL-17 receptors. (C) Histogram displaying IL-13 receptor mRNA levels (IL4R, IL2RG, IL13RA1, and IL13RA2) in epithelial cells after 12 h of stimulation with OSM (n = 4). (D) Scatter plot displaying the correlation between the IL4R expression level in epithelial cells and the OSM total expression level in the scRNA-sequencing data of polyp samples. (E-F) Histogram displaying the G-CSF and IL-8 mRNA expression levels after 12 h of stimulation with OSM and IL-17A in fibroblasts (n = 6). (G-H) Histogram displaying the G-CSF and IL-8 mRNA expression levels after 12 h of stimulation with OSM and IL-17A in HNECs (n = 6). (I) Histogram displaying the IL-17 receptor mRNA levels (IL17RA and IL17RC) in fibroblasts (n =

4) and HNECs (n = 4). * $p<0.05$, ** $p<0.01$, *** $p<0.001$, **** $p<0.001$.

Supplemental Tables

Supplemental Table 1 Characteristics of the subjects

Supplemental Table 2 Filtered genes in scRNA-sequencing

Supplemental Table 3 Marker genes of scRNA-sequencing

Supplemental Table 4 Primers used for quantitative PCR analysis

Supplemental Table 5 Antibodies in flow cytometry

Supplemental Table 6 The differential expressed genes among cell types

Supplemental Table 7 The differential gene expression based on trajectory construction and pseudotime analyses

Supplemental Table 8 The differential expressed genes of neutrophils between the neCRSwNP and the PB group

Supplemental Table 9 The differential expressed genes of neutrophils between the eCRSwNP and the PB group

Supplemental Table 10 The gene ontology (GO) enrichment analysis of neutrophils between the eCRSwNP and the PB group

Supplemental Table 11 The Kyoto encyclopedia of genes and genomes (KEGG) enrichment analysis of neutrophils between the eCRSwNP and the PB group

Supplemental Table 12 The gene set enrichment analysis (GSEA) of neutrophils between the eCRSwNP and the PB group

Supplemental Table 13 The gene ontology (GO) enrichment analysis of neutrophils between the neCRSwNP and the PB group

Supplemental Table 14 The Kyoto encyclopedia of genes and genomes (KEGG) enrichment analysis of neutrophils between the neCRSwNP and the PB group

Supplemental Table 15 The gene set enrichment analysis (GSEA) of neutrophils between the neCRSwNP and the PB group

Supplemental Table 16 The differential gene expression among neutrophil clusters

Supplemental Table 17 The differential gene expression among neutrophil subsets

Supplemental Table 18 The transcription factor analysis of neutrophil subsets

Supplemental Table 19 The differential gene expression among groups in S100A8+ neutrophils

Supplemental Table 20 The differential gene expression among groups in GBP5+ neutrophils

Supplemental Table 21 The differential gene expression among groups in CXCL8+ neutrophils

Supplemental Table 22 The differential gene expression among groups in EPHB1+ neutrophils

Supplemental Table 23 The differential gene expression among groups in S100A12+ neutrophils
